# Abiotic and habitat drivers of tick vector abundance, diversity, phenology and human encounter risk in southern California

**DOI:** 10.1371/journal.pone.0201665

**Published:** 2018-07-31

**Authors:** Andrew J. MacDonald

**Affiliations:** 1 Department of Biology, Stanford University, Stanford, California, United States of America; 2 Earth Research Institute, University of California, Santa Barbara, California, United States of America; University of Minnesota, UNITED STATES

## Abstract

The distribution, abundance and seasonal activity of vector species, such as ticks and mosquitoes, are key determinants of vector-borne disease risk, and are strongly influenced by abiotic and habitat conditions. Despite the numerous species of tick vectors in the heavily populated North American West Coast, all but *Ixodes pacificus*, the primary vector of the Lyme disease spirochete, is poorly characterized with regard to seasonal activity patterns and fine scale drivers of distribution and abundance, particularly in heavily populated regions of southern California. This lack of knowledge inhibits both scientific understanding and public health efforts to minimize vector exposure and risk of pathogen transmission to humans. Here we address this gap by characterizing the abiotic and habitat drivers of the distribution, abundance, and diversity of the vector tick community using fine scale temporal surveys over two seasons (2014 and 2015) across coastal and inland regions of Santa Barbara County, CA. We also characterize patterns of seasonal activity of the more common vector species to understand seasonality in risk of vector exposure, and specifically focus on human encounter risk using standardized tick drags as our method of collection. Leveraging plot-level habitat and abiotic variables in partial least squares regression analysis, we find the seven different vector species collected in this study have divergent drivers of activity and abundance. For example, *I*. *pacificus* is strongly associated with dense forest habitats and cool and moist microclimates, while *Dermacentor occidentalis* and *Dermacentor variabilis*, competent vectors of Rocky Mountain Spotted Fever, were found to be more tolerant of higher average temperatures and more open habitats. These results suggest that *I*. *pacificus* may be expected to experience reductions in geographic distribution and seasonal activity under projected land cover and climate change in coastal southern California, while *D*. *occidentalis* may experience more limited effects. We discuss implications for changing tick-borne disease risk associated with pathogens transmitted by *Ixodes* as well as *Dermacentor* species ticks in the western US, and contrast these predictions with eastern North America.

## Introduction

The recent emergence and spread of numerous vector-borne zoonotic diseases, including West Nile virus, Zika virus and Lyme disease, has been broadly attributed to processes of global change that influence the distribution and ecological dynamics of host and vector species [1–4]. Maintained in complex ecological systems, transmission of vector-borne zoonoses depends on interaction between hosts, pathogens and vectors, which is strongly influenced by the environment [2,5]. For example, vector and pathogen development may be temperature dependent [6] or require specific or ephemeral habitats [7], while hosts may be limited by resource availability [8], driven by habitat or weather conditions. Further, abiotic conditions can also be important determinants of seasonal activity patterns or phenology of hosts and vectors [9–11], which determines the degree of interaction between key host and vector species, influencing pathogen transmission dynamics [12].

Understanding the habitat associations and abiotic determinants of vector and host distribution, as well as the seasonal activity patterns of important vector species, is key to determining risk of vector exposure and pathogen transmission. Moreover, this information is crucial to predicting how the distribution of both disease vectors and human disease risk are likely to change under processes of global change, such as climate and land use change.

In the northern hemisphere, tick-borne diseases are the most common vector-borne diseases of humans, and ticks transmit one of the most diverse arrays of pathogens of any vector taxanomic group. For example, in North America, *Ixodes* species ticks alone transmit a diversity of bacteria (e.g. *Borrelia burgdorferi*—causative agent of Lyme disease), viruses (e.g. Powassan virus) and protozoans (e.g. *Babesia* species—causative agents of babesiosis) that cause disease in humans. Other tick species with different ecologies also transmit a diversity of pathogens from spotted fever group rickettsiae to *Francisella tularensis* (causative agent of tularemia), transmitted by *Dermacentor* species ticks [13]. Numerous host-specialist tick vectors are thought to play an important role in the maintenance of enzootic pathogen transmission cycles in vertebrate host communities, which may influence the likelihood of zoonotic spillover into human populations [14,15]. Studies focusing on a single tick vector species may not be sufficient to capture the aggregate effects of climate or land use and land cover on disease risk. This also may not capture key shifts in vector seasonality or distribution that both influence vector exposure risk as well as the proper allocation of control measures and public health intervention.

The majority of studies investigating tick vector ecology and phenology [9,10,16,17] in California have focused on the Western blacklegged tick (*Ixodes pacificus*), the primary vector of the Lyme disease spirochete in the western US. While broad patterns of distribution of *I*. *pacificus* have been characterized in the United States, for example at the county level [18] and using species distribution models to investigate climate suitability in California [19], fine scale information about the abiotic drivers and habitat associations of vector tick communities is lacking, particularly in understudied and densely populated regions like central and southern coastal California. There are numerous other species of ticks of medical and veterinary importance that transmit human pathogens throughout the state [20], with some playing a more prominent role as vectors in some regions than others. For example, while infection prevalence with *Borrelia burgdorferi* in *I*. *pacificus* is higher in the northwestern region of the state [21], infection with *Rickettsia philippi*, the causative agent of Pacific Coast tick fever, in *Dermacentor occidentalis* ticks appears to be higher in southern California [22]. Aggregate tick-borne disease risk depends on the ecology of the community of tick vectors, and will respond differently to processes of global change depending on the region and the vector species.

In this study, we investigate and characterize the patterns of seasonal activity, and habitat and abiotic drivers of the distribution, abundance and diversity of the community of vector ticks in coastal southern California. Habitats and microclimates are highly heterogeneous and diverse, and can vary substantially across small spatial scales in California. We focus on identifying where and when human risk of vector tick exposure is highest in diverse habitats of coastal and inland Santa Barbara County, California using high temporal and spatial resolution, standardized tick drag sampling. Such a sampling approach is necessary to identify how risk of exposure might change across highly diverse landscapes. Specifically, we investigate 1) whether the distribution and abundance of medically important tick vectors respond similarly to abiotic and habitat conditions? 2) whether vector species thought to be involved in enzootic pathogen transmission cycles share environmental drivers with the vector species involved in zoonotic transmission of these pathogens? and 3) discuss how key vectors of human pathogens are likely to respond to future changes in climate and land cover. Given low rates of infection with the causative agent of Lyme disease in *I*. *pacificus* populations in southern California from earlier studies [23–26], we expect to find that *I*. *pacificus* and possible enzootic vectors of the Lyme spirochete do not share abiotic drivers and display limited temporal (seasonal activity patterns) and spatial (habitat preferences) overlap in this region of southern California. In contrast, with elevated transmission activity of spotted fever group rickettsiae in southern California [22], we expect to find greater spatial and temporal overlap between zoonotic and possible enzootic vectors, and broader distribution and habitat associations of these species in the study region.

## Materials and methods

**Fig 1:**
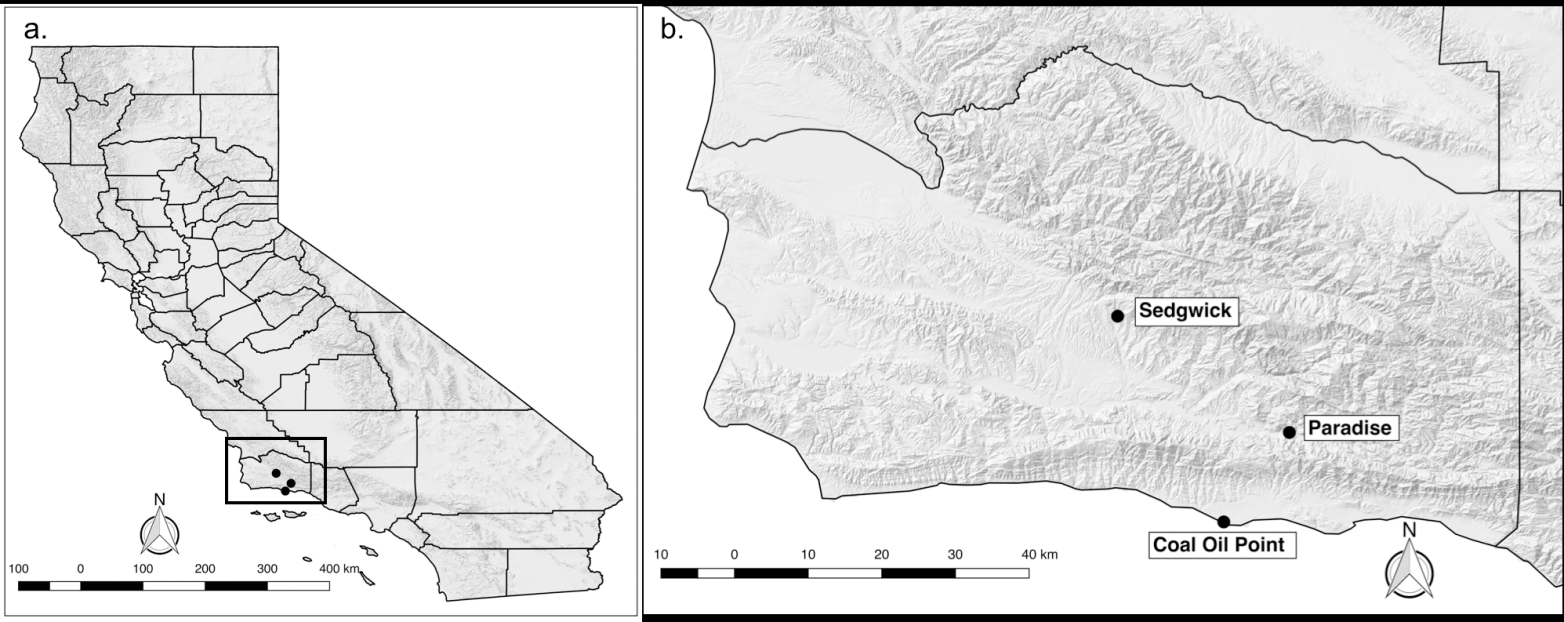
Map of study sites. a) Location of sites in the context of the state of California, and b) specific location within Santa Barbara County of each sampled reserve.

This study was conducted in Santa Barbara County, California in three ecological reserves representative of the diversity of habitats and climates found in coastal central and southern California (S1 and S2 Figs). Santa Barbara County, and the populated regions of coastal California broadly, has a Mediterranean climate with relatively cool, wet winters and warm, dry summers (S1 Fig). Field sampling was conducted in: 1) Sedgwick Reserve (34°42’04.38”N, 120°02’50.81”W), an inland reserve administered by the University of California Natural Reserve System (UCNRS) and located in the Santa Ynez Valley, which is dominated by grassland, oak woodland and oak savannah habitats; 2) Paradise Reserve (34°32’22.07”N, 119°47’51.89”W), a privately owned reserve located on the north side of the Santa Ynez Mountains, which is dominated by dense oak woodland, riparian and chaparral habitats; and 3) Coal Oil Point Reserve (34°24’52.96”N, 119°52’48.59”W), a coastal reserve administered by the UCNRS, which is dominated by coastal sage scrub, grassland and coast live oak habitats. Permission to access and use Sedgwick Reserve and Coal Oil Point Reserve field sites was issued by the University of California Natural Reserve System. Permission to access and use Paradise Reserve field site was issued by the owners, Dr. Cris Sandoval and Dr. Kevin Lafferty. No endangered or protected species were used in this study. While Sedgwick and Paradise Reserves are characteristic of inland, wildland habitats in the region, Coal Oil Point Reserve is located in a suburban, coastal setting characteristic of natural habitats found in the more densely populated coastal regions of the state (Fig 1).

### Tick collection and characterization of habitat and abiotic conditions

Within each reserve, 50x50m subplots were established in which the tick community was sampled, 10 each in Sedgwick and Coal Oil Point Reserves, and 4 in Paradise Reserve for a total of 24 sampled subplots. Subplots were selected in a stratified random manner, to ensure comparable coverage of each broad habitat type across the three reserves (e.g. an equal number of plots dominated by grassland, shrub/chaparral, oak savannah and oak woodland habitats). Fewer plots were sampled in Paradise Reserve to prevent oversampling of any particular habitat across the study region, due to the comparative homogeneity in habitat types in this reserve (e.g. oversampling dense oak woodland due to over-representation of this habitat type in Paradise Reserve). To determine vector tick seasonality by life stage, standardized drag sampling, in which a 1x1m white flannel cloth is dragged along the ground and understory vegetation and attached ticks collected, was undertaken approximately every 7–14 days in each subplot from December 2013 to June 2015, encompassing two seasons of winter and spring activity, and one full summer season. Standardized 50m transects were sampled in each subplot on each sampling event, and a total area of 500m^2^ was sampled per subplot, per sampling event. Flags were checked for ticks every 10m, or 5 times per transect. All life stages of all hard ticks were collected, preserved in 70% ethanol and returned to the lab for species identification using a dichotomous key for the ticks of California [27]. Seasonality and distribution data from drag sampling was then compared to data on tick host feeding collected from each of the three reserves from previously published studies [24] and unpublished work.

Within each subplot, data on habitat characteristics and environmental conditions were collected, and the most relevant variables to tick vector ecology chosen based on previous studies [11,16,17,23,28]. These included average maximum daily temperature in the wet (November 1 –April 30) and dry (May 1 –October 31) seasons using iButton (Maxim Integrated, San Jose, CA) data loggers placed at the ground surface in each subplot, vegetation metrics including canopy cover, stem density (number of stems > 5cm diameter at breast height and > 1.5m in height), percent cover of leaf litter, bare ground, herbaceous cover and understory woody vegetation, as well as slope and elevation. All plot-level habitat data were collected during the middle of the wet season in April of each year (April 2014 and 2015), while plot-level temperature data were collected continuously throughout the study. These standardized plot characteristics were used to determine the habitat and abiotic drivers of the distribution and abundance of each vector species, but were not used to investigate environmental drivers of tick phenology. The distribution and abundance of host species are also key factors in determining the distribution and abundance of vector ticks. However, a comprehensive assessment of host abundance, by species and by individual sample plot, was outside the scope of the present study. Instead, this analysis focused on abiotic and habitat drivers, which themselves are important determinants of the distribution of key host species for ticks.

### Statistical analysis

Partial least squares regression (PLSR) models were fit using the plot-level habitat and abiotic independent variables described above to predict average and peak abundance of each vector tick species encountered during weekly to bimonthly drag sampling. PLSR is a method of data dimensionality reduction similar to principal components analysis (PCA). It is particularly well suited to this context because, relative to sample size, there are a large number of highly correlated (collinear) abiotic and habitat predictor variables thought to influence tick vector distribution and population dynamics. The method combines features of PCA and multiple regression to eliminate multicollinearity in the independent variables that would otherwise bias the multiple regression approach, as well as address the problem of choosing an optimum subset of predictors that remains in PCA regression approaches [23,29]. As in PCA, a set of orthogonal latent vectors comprised of correlated predictor variables are constructed and those latent vectors are used as predictors in a subsequent regression step to eliminate highly correlated independent variables [29]. However, unlike in PCA where orthogonal latent vectors are constructed that explain as much of the *variance* in the independent variables as possible, in PLSR the outcome variable (tick abundance in this study) is included in the construction of latent vectors. Latent vectors are then chosen that explain as much of the *covariance* between the independent and dependent variables as possible, ensuring the relevance of the latent vectors as predictors in the regression.

PLSR models were run independently for each tick species and each life stage (with the exception of species/life stages which were not detected, or had only a single observation), with annual plot-level average and peak abundance as the dependent outcome variables, and the set of abiotic and habitat variables as independent predictor variables. Peak and average abundance were highly correlated within species and life stage, thus multivariate models were run with both peak and average density as outcome variables for each species-life stage combination. An additional univariate model was specified with tick diversity (Shannon’s H) as the outcome variable with the same set of abiotic and habitat predictor variables. All statistical analyses and data processing were conducted in R 3.4.2 [30], and PLSR models were run using the package ‘*plsdepot*’ [31].

## Results

### Tick vector phenology

In total, 7 different species of tick vector were collected over the course of this study including *Ixodes pacificus* (Western blacklegged tick), *Dermacentor occidentalis* (Pacific Coast tick), *Dermacentor variabilis* (American dog tick), *Haemaphysalis leporispalustris* (rabbit tick), *Ixodes brunneus*, *Ixodes spinipalpis* and *Ixodes peromysci* (Table 1). Some of these species, notably *I*. *peromysci* and *I*. *spinipalpis*, were comparatively rarely encountered throughout the study region. In addition, some species, including *H*. *leporispalustris* and *D*. *variabilis*, had some life stages that were commonly encountered and others that were not. These species- and life stage-specific patterns of abundance could be due to differential host-seeking behavior (e.g. endophilic vs. exophilic), and thus likelihood of collection, or may represent true differences in abundance.

**Table 1:** Total number of ticks collected by species and life stage.

Species	Life stage		
	Adults	Nymphs	Larvae
*I*. *pacificus*	292	67	410
*D*. *occidentalis*	315	22	194
*D*. *variabilis*	439	0	96
*H*. *leporispalustris*	1	53	209
*I*. *brunneus*	6	8	176
*I*. *spinipalpis*	1	36	7
*I*. *peromysci*	1	5	0

**Fig 2:**
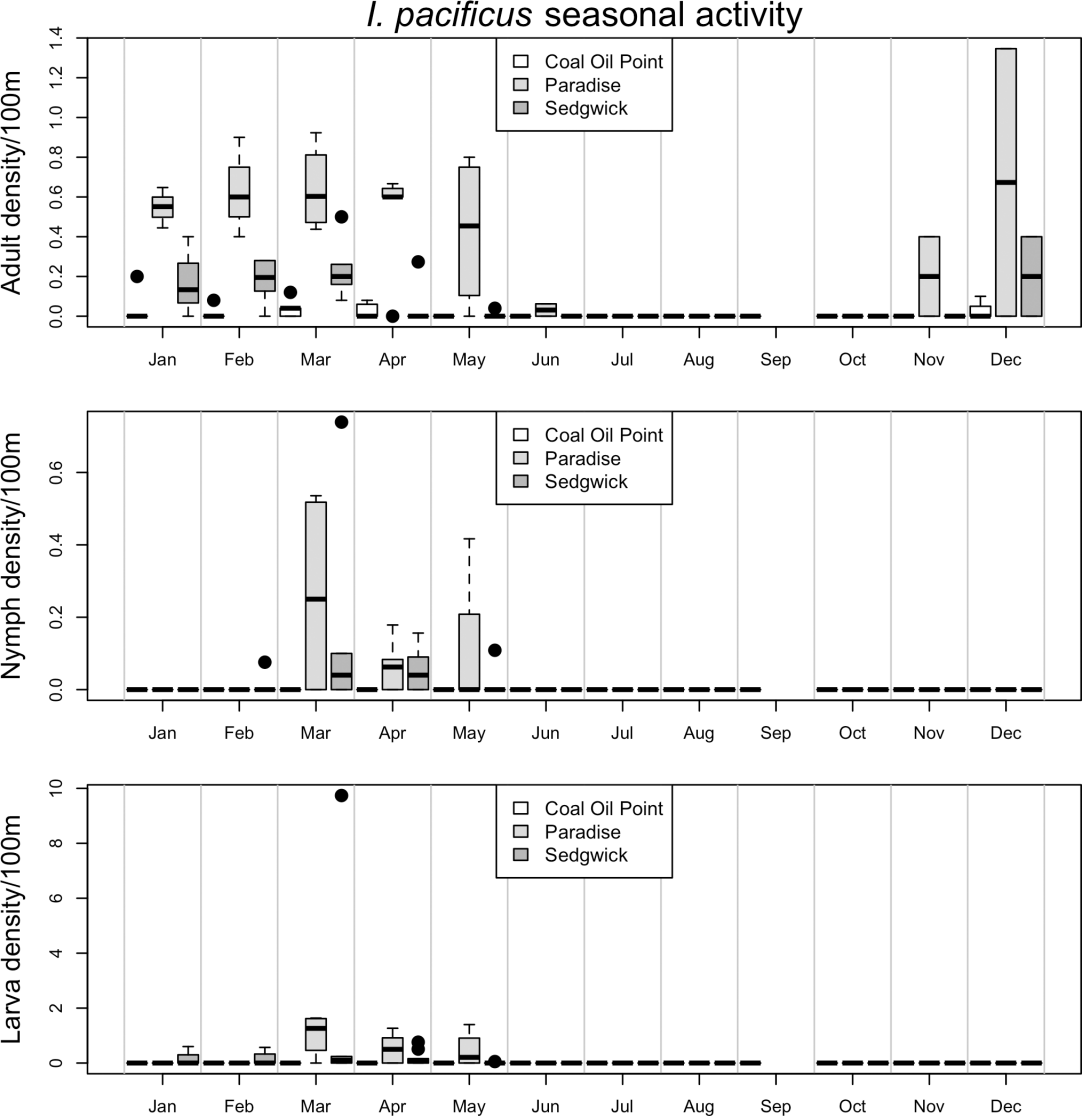
Seasonal activity of *I*. *pacificus*. Represented as density of ticks per 100m^2^ by month. Adults are in the top panel, nymphs in the middle and larvae on the bottom. The three sites, *Coal Oil Point* (white), *Paradise* (light grey) and *Sedgwick* (dark grey), are represented by individual bars in each month, in that order. Black dots represent outliers; horizontal bars in box plots represent the mean, while boxes and whiskers represent quartiles; horizontal bars without box plots represent a single sample in which that species/life stage was collected in a given month; horizontal bars at ‘0’ on the y-axis represent months in which collection occurred, but no ticks were collected.

**Fig 3:**
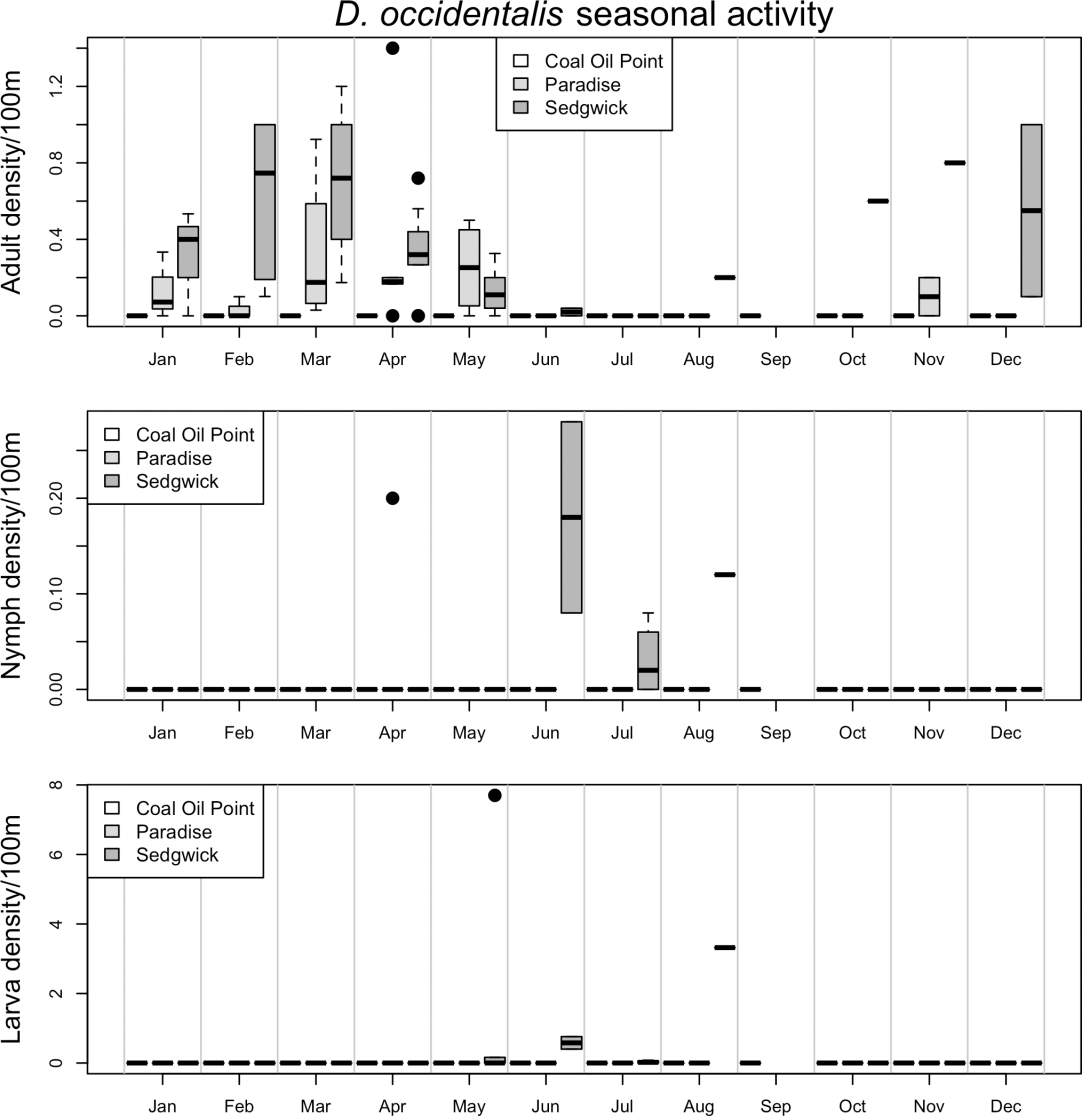
Seasonal activity of *D*. *occidentalis*. Represented as density of ticks per 100m^2^ by month. Adults are in the top panel, nymphs in the middle and larvae on the bottom. The three sites, *Coal Oil Point* (white), *Paradise* (light grey) and *Sedgwick* (dark grey), are represented by individual bars in each month, in that order. Black dots represent outliers; horizontal bars in box plots represent the mean, while boxes and whiskers represent quartiles; horizontal bars without box plots represent a single sample in which that species/life stage was collected in a given month; horizontal bars at ‘0’ on the y-axis represent months in which collection occurred, but no ticks were collected.

**Fig 4:**
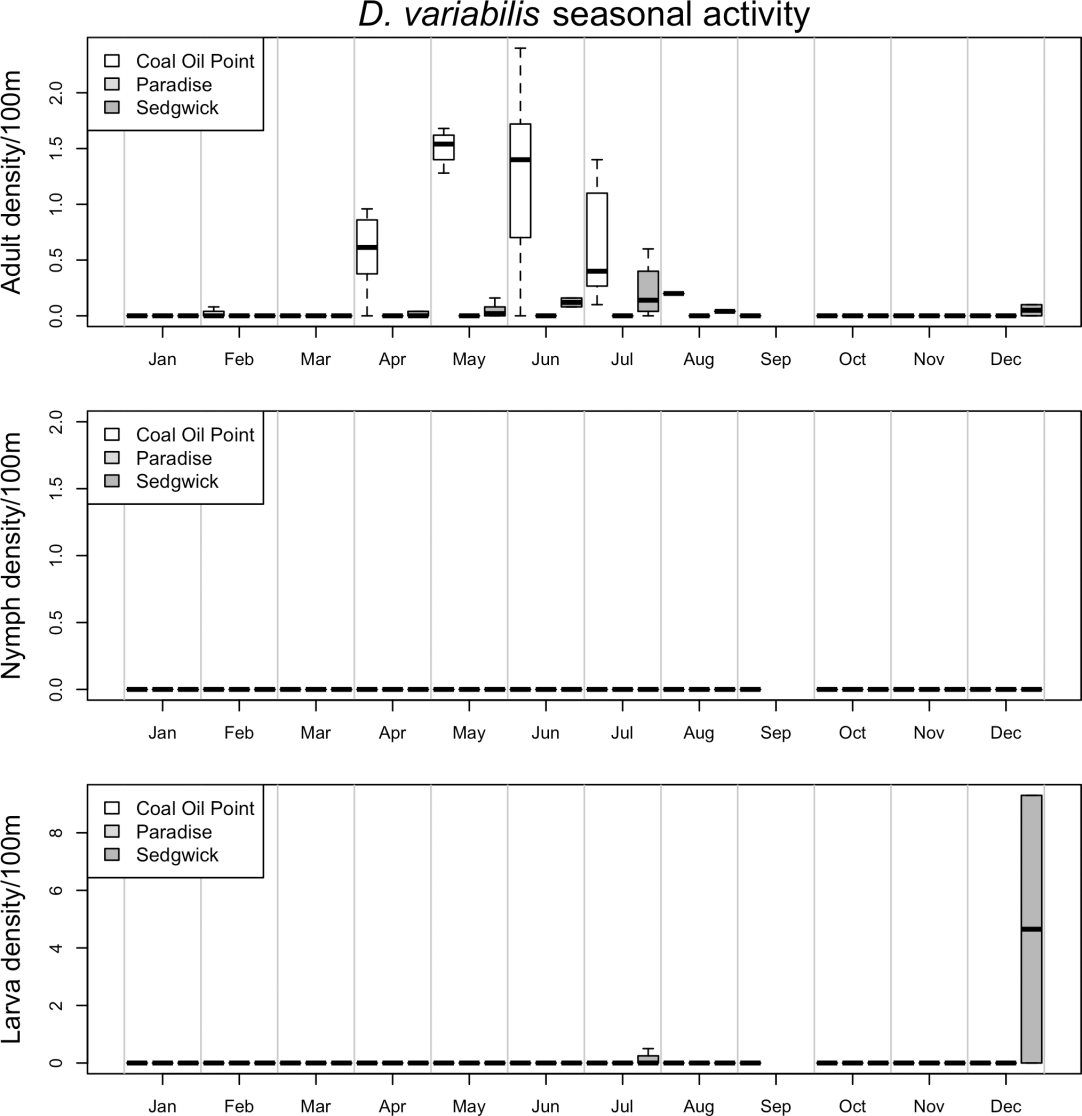
Seasonal activity of *D*. *variabilis*. Represented as density of ticks per 100m^2^ by month. Adults are in the top panel, nymphs in the middle and larvae on the bottom. The three sites, *Coal Oil Point* (white), *Paradise* (light grey) and *Sedgwick* (dark grey), are represented by individual bars in each month, in that order. Black dots represent outliers; horizontal bars in box plots represent the mean, while boxes and whiskers represent quartiles; horizontal bars without box plots represent a single sample in which that species/life stage was collected in a given month; horizontal bars at ‘0’ on the y-axis represent months in which collection occurred, but no ticks were collected.

**Fig 5:**
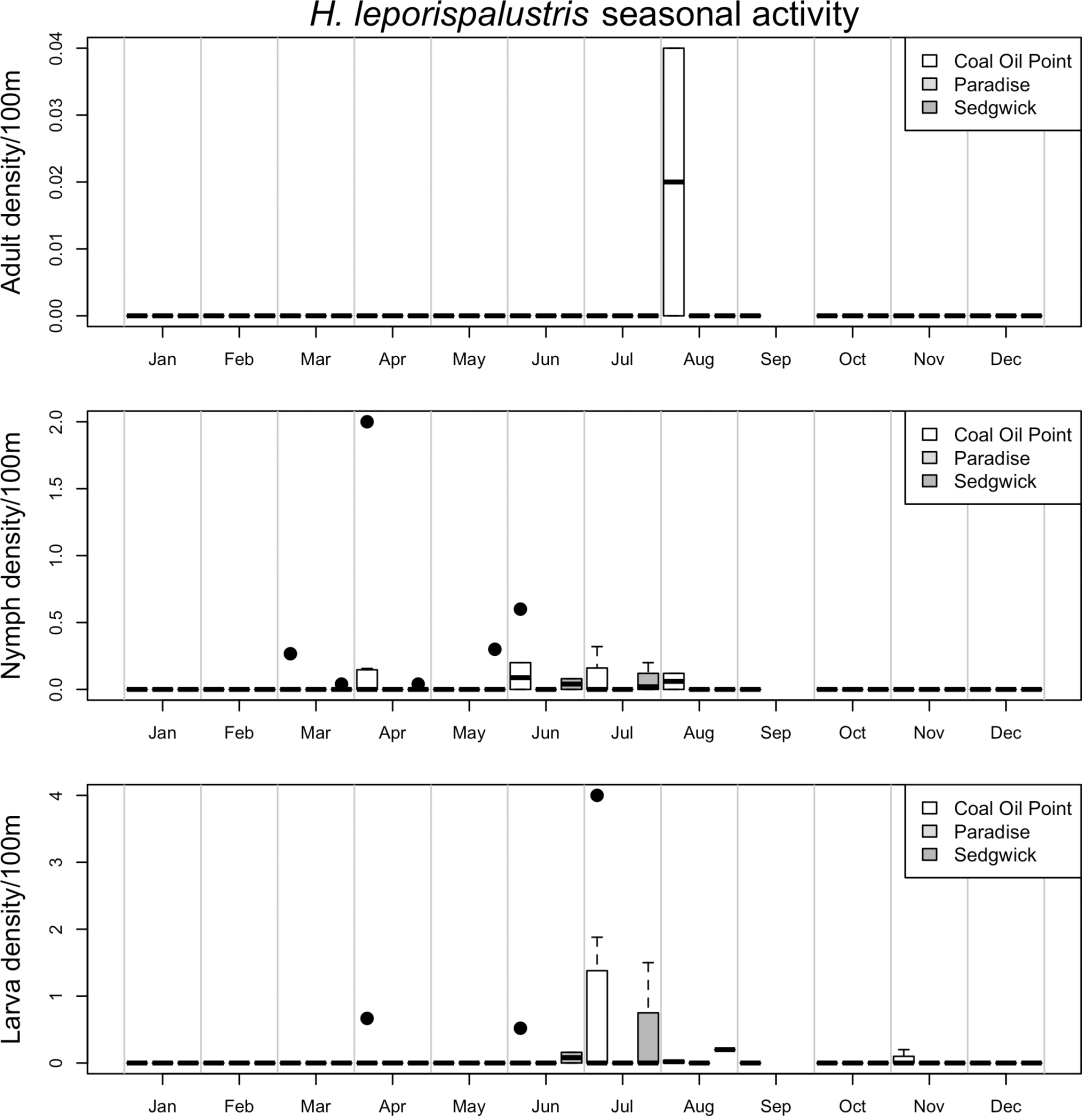
Seasonal activity of *H*. *leporispalustris*. Represented as density of ticks per 100m^2^ by month. Adults are in the top panel, nymphs in the middle and larvae on the bottom. The three sites, *Coal Oil Point* (white), *Paradise* (light grey) and *Sedgwick* (dark grey), are represented by individual bars in each month, in that order. Black dots represent outliers; horizontal bars in box plots represent the mean, while boxes and whiskers represent quartiles; horizontal bars without box plots represent a single sample in which that species/life stage was collected in a given month; horizontal bars at ‘0’ on the y-axis represent months in which collection occurred, but no ticks were collected.

**Fig 6:**
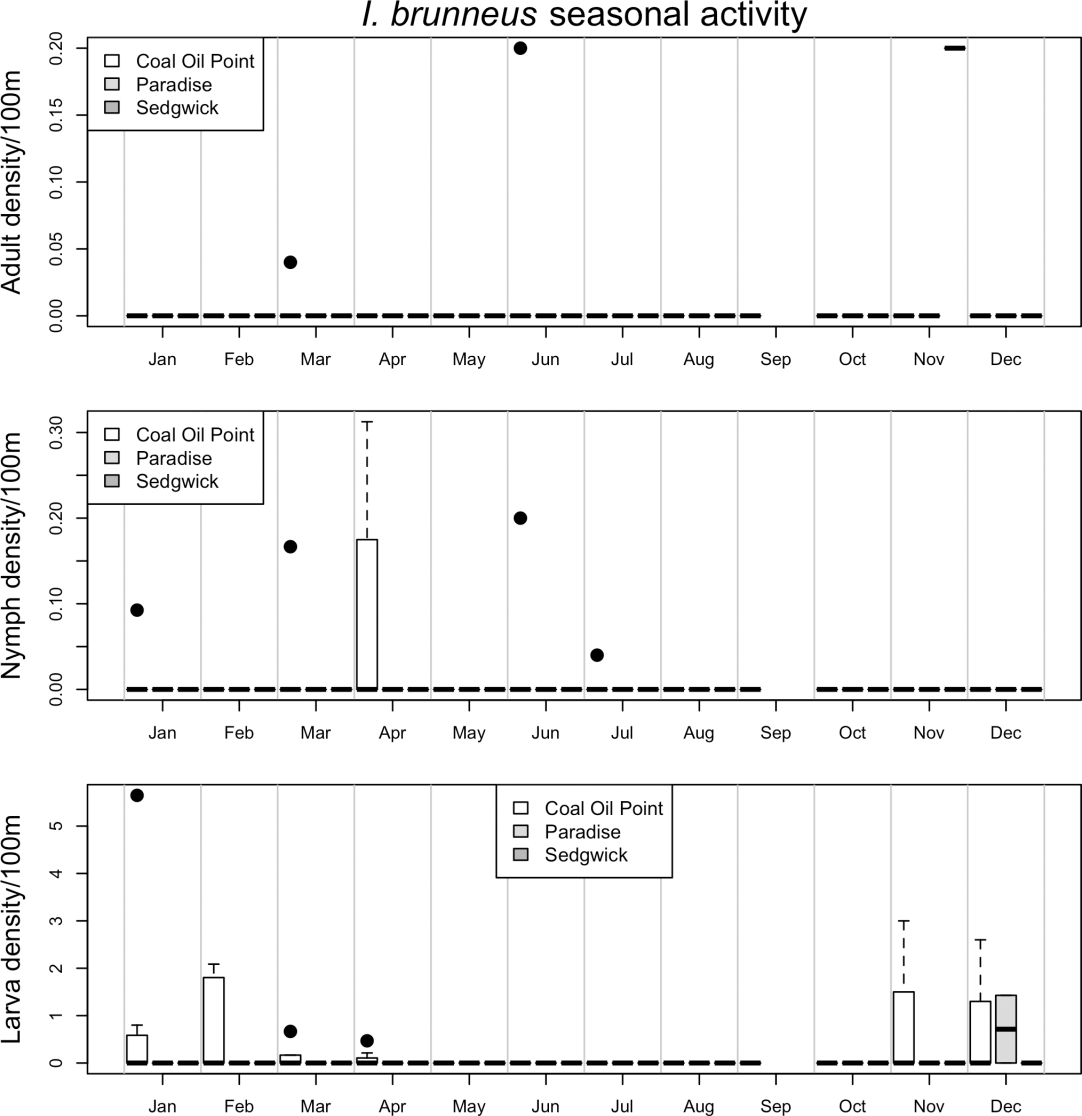
Seasonal activity of *I*. *brunneus*. Represented as density of ticks per 100m^2^ by month. Adults are in the top panel, nymphs in the middle and larvae on the bottom. The three sites, *Coal Oil Point* (white), *Paradise* (light grey) and *Sedgwick* (dark grey), are represented by individual bars in each month, in that order. Black dots represent outliers; horizontal bars in box plots represent the mean, while boxes and whiskers represent quartiles; horizontal bars without box plots represent a single sample in which that species/life stage was collected in a given month; horizontal bars at ‘0’ on the y-axis represent months in which collection occurred, but no ticks were collected.

Seasonal patterns of activity differed markedly between vector species and life stages (Figs 2–6, S3 and S4). For example, while the adult stage of the two most common tick species in this study (*I*. *pacificus* and *D*. *occidentalis*) were primarily active in the winter and spring months, the juvenile stages of *I*. *pacificus* displayed a narrow range of springtime activity while juvenile *D*. *occidentalis* were more often encountered in the summer months (Figs 2 and 3). There are also striking differences in abundance between sampled sites, for example, all life stages of *I*. *pacificus* were more abundant in the cooler and wetter Paradise Reserve, while all stages of *D*. *occidentalis* were more abundant in the hotter and drier Sedgwick Reserve (Figs 2 and 3).

*D*. *variabilis* adults were most active in the late spring and summer, and primarily at the more temperate Coal Oil Point Reserve, while the juvenile stages were encountered primarily in the winter months (Fig 4). All life stages of *H*. *leporispalustris* were primarily active in the late spring and summer, and were most abundant in Coal Oil Point Reserve (Fig 5). Juvenile *I*. *brunneus* were most commonly encountered in the early winter through the spring months, while the adults were not common enough to distinguish a clear seasonal pattern (Fig 6). Finally, *I*. *spinipalpis* and *I*. *peromysci* did not display strong seasonality due to low capture rates, with the exception that they were generally not encountered during the hottest summer months, from June through September (S3 and S4 Figs). This was true of all life stages of these tick species, which were much more abundant at the temperate Coal Oil Point Reserve than either of the inland sites.

While observations of seasonal activity and broad patterns of geographic distribution of some species appear to be well characterized by drag sampling, for other species and life stages drag sampling may underestimate abundance and poorly characterize seasonal activity (S5–S12 Figs) [24]. For example, seasonal activity and geographic distribution of *I*. *pacificus*, *D*. *occidentalis*, *D*. *variabilis* and *I*. *spinipalpis* as determined by drag sampling closely matches what is observed in data on patterns of host feeding on small vertebrate hosts across the three reserves (S5–S8 Figs and S1 Table). However, drag sampling appears to underestimate abundance and poorly characterize seasonal activity of the juvenile stages of *D*. *variabilis*, *I*. *spinipalpis* and *I*. *peromysci* relative to host sampling (S7, S8, S10 and S12 Figs). Finally, some species (e.g. *I*. *brunneus* and *H*. *leporispalustris*) have more specialist host-feeding preferences, on birds and lagomorphs respectively, and were poorly characterized by small vertebrate host sampling in this study (S9, S11 and S12 Figs), but appear to be fairly well characterized by drag sampling (Figs 5 and 6).

### Abiotic and habitat drivers of tick distribution, abundance and diversity

Mirroring divergent patterns of seasonal activity, the abundance and distribution of different tick species were found to be associated with different habitat types and abiotic conditions. The results of each PLSR model, run for each individual tick species and life stage, are presented in Tables 2–5. Included in each model output are two extracted latent vectors, or components, each accounting for some proportion (R-squared) of the overall covariance between the predictor variables and the outcome variables (average and peak tick abundance). Variable weights and variable importance in the projection (VIP) scores indicate the direction and strength of contribution of each variable to the relationship, respectively [29]. The sum of squared variable weights for each latent vector is equal to one, thus the overall contribution of each variable to the interpretation and meaning of each latent vector can be estimated. Additionally, a VIP score above 1 indicates a significant contribution of that variable to the relationship between tick abundance and abiotic and habitat predictors, and suggests an important role of that variable in driving average and peak vector tick abundance [29].

**Table 2:** PLSR model results for abundance of *I*. *pacificus* and *D*. *occidentalis*.

Variable	*Ixodes pacificus*						*Dermacentor occidentalis*					
	Adults		Nymphs		Larvae		Adults		Nymphs		Larvae	
	Wts. (VIP) Comp. 1	Wts. (VIP) Comp. 2	Wts. (VIP) Comp. 1	Wts. (VIP) Comp. 2	Wts. (VIP) Comp. 1	Wts. (VIP) Comp. 2	Wts. (VIP) Comp. 1	Wts. (VIP) Comp. 2	Wts. (VIP) Comp. 1	Wts. (VIP) Comp. 2	Wts. (VIP) Comp. 1	Wts. (VIP) Comp. 2
Site	0.59 (1.95)	0.51 (1.84)	0.40 (1.31)	0.01 (1.16)	0.27 (0.88)	-0.35 (0.98)	0.42 (1.39)	-0.12 (1.23)	0.30 (1.01)	-0.28 (0.98)	0.16 (0.52)	-0.55 (1.13)
Avg. Summer Temp.	-0.16 (0.54)	0.08 (0.44)	-0.14 (0.47)	0.09 (0.44)	-0.09 (0.29)	0.30 (0.64)	0.30 (1.00)	0.46 (1.15)	0.12 (0.40)	0.17 (0.47)	0.25 (0.84)	0.29 (0.88)
Avg. Winter Temp.	-0.37 (1.23)	-0.29 (1.12)	-0.17 (0.55)	0.20 (0.58)	-0.12 (0.40)	0.35 (0.77)	0.27 (0.91)	0.62 (1.28)	0.39 (1.29)	0.72 (1.75)	0.23 (0.75)	0.43 (1.02)
Elevation	0.33 (1.10)	-0.17 (0.90)	0.33 (1.10)	-0.22 (1.02)	0.32 (1.05)	-0.17 (0.91)	0.58 (1.93)	0.07 (1.69)	0.56 (1.86)	0.00 (1.51)	0.39 (1.30)	-0.28 (1.19)
Slope	0.14 (0.45)	-0.57 (1.31)	0.13 (0.42)	-0.65 (1.07)	0.19 (0.63)	-0.35 (0.85)	0.27 (0.91)	-0.18 (0.85)	0.37 (1.22)	-0.06 (0.99)	0.10 (0.32)	-0.41 (0.82)
Canopy Cover	0.36 (1.19)	-0.26 (1.05)	0.38 (1.27)	-0.22 (1.17)	0.35 (1.16)	-0.29 (1.09)	0.26 (0.86)	-0.36 (0.95)	0.17 (0.55)	-0.50 (1.07)	0.38 (1.26)	-0.14 (1.07)
Leaf Litter Cover	0.25 (0.82)	-0.37 (1.03)	0.45 (1.48)	0.18 (1.34)	0.60 (1.97)	0.53 (1.90)	0.22 (0.74)	-0.23 (0.74)	0.32 (1.07)	-0.12 (0.89)	0.38 (1.26)	0.03 (1.04)
Shrub Cover	0.02 (0.07)	0.20 (0.45)	0.02 (0.08)	0.16 (0.25)	0.05 (0.18)	0.11 (0.26)	-0.25 (0.83)	0.07 (0.73)	-0.28 (0.94)	0.00 (0.76)	-0.42 (1.38)	-0.15 (1.17)
Herbaceous Cover	-0.14 (0.46)	-0.02 (0.34)	-0.26 (0.87)	-0.33 (0.92)	-0.33 (1.09)	-0.35 (1.11)	0.12 (0.38)	0.05 (0.35)	0.10 (0.35)	0.05 (0.30)	0.15 (0.48)	0.07 (0.42)
Bare Ground Cover	0.03 (0.09)	0.05 (0.14)	0.19 (0.64)	0.52 (0.99)	0.03 (0.09)	0.04 (0.12)	0.07 (0.23)	-0.04 (0.21)	0.06 (0.18)	0.01 (0.15)	0.33 (1.10)	0.30 (1.06)
Stem Density	0.39 (1.29)	-0.22 (1.07)	0.47 (1.55)	0.03 (1.37)	0.43 (1.43)	-0.13 (1.18)	0.22 (0.74)	-0.40 (0.91)	0.26 (0.85)	-0.33 (0.94)	0.33 (1.08)	-0.19 (0.96)
R-squared	0.360	0.294	0.464	0.129	0.393	0.214	0.502	0.152	0.257	0.135	0.251	0.119
Cumulative R-squared	0.654		0.593		0.607		0.654		0.392		0.370	

Variable weights (wts.) indicate the direction of the effect and VIP scores > 1 indicate a significant contribution of that variable to the variation explained by each component. Variables explaining a significant amount of the variation in the first component are highlighted in blue (positive) and red (negative).

**Table 3:** PLSR model results for abundance of *D*. *variabilis* and *H*. *leporispalustris*.

Variable	*Dermacentor variabilis*						*Haemaphysalis leporispalustris*					
	Adults		Nymphs		Larvae		Adults		Nymphs		Larvae	
	Wts. (VIP) Comp. 1	Wts. (VIP) Comp. 2	Wts. (VIP) Comp. 1	Wts. (VIP) Comp. 2	Wts. (VIP) Comp. 1	Wts. (VIP) Comp. 2	Wts. (VIP) Comp. 1	Wts. (VIP) Comp. 2	Wts. (VIP) Comp. 1	Wts. (VIP) Comp. 2	Wts. (VIP) Comp. 1	Wts. (VIP) Comp. 2
Site	-0.45 (1.49)	-0.11 (1.41)	NA	NA	0.14 (0.45)	-0.36 (0.97)	NA	NA	-0.47 (1.54)	0.11 (1.36)	-0.38 (1.25)	0.18 (1.05)
Avg. Summer Temp.	0.02 (0.06)	-0.23 (0.26)	NA	NA	-0.08 (0.25)	0.10 (0.29)	NA	NA	-0.39 (1.28)	-0.42 (1.31)	0.36 (1.18)	0.31 (1.13)
Avg. Winter Temp.	0.03 (0.09)	-0.52 (0.57)	NA	NA	-0.04 (0.13)	0.28 (0.72)	NA	NA	-0.07 (0.23)	-0.13 (0.29)	0.22 (0.74)	-0.02 (0.58)
Elevation	-0.49 (1.63)	-0.16 (1.55)	NA	NA	0.30 (0.99)	-0.26 (0.91)	NA	NA	-0.46 (1.51)	0.21 (1.36)	-0.40 (1.31)	0.15 (1.08)
Slope	-0.32 (1.07)	0.11 (1.02)	NA	NA	0.76 (2.52)	0.22 (1.70)	NA	NA	-0.28 (0.93)	0.10 (0.83)	-0.08 (0.25)	0.46 (0.96)
Canopy Cover	-0.38 (1.26)	0.21 (1.21)	NA	NA	0.45 (1.48)	-0.09 (0.97)	NA	NA	-0.23 (0.75)	0.37 (0.89)	-0.18 (0.58)	0.41 (0.96)
Leaf Litter Cover	-0.25 (0.83)	0.37 (0.88)	NA	NA	-0.04 (0.13)	-0.54 (1.38)	NA	NA	0.11 (0.36)	0.61 (1.04)	-0.03 (0.09)	0.42 (0.87)
Shrub Cover	0.26 (0.87)	0.30 (0.88)	NA	NA	-0.12 (0.39)	0.16 (0.49)	NA	NA	0.16 (0.52)	-0.23 (0.58)	0.50 (1.65)	0.13 (1.32)
Herbaceous Cover	-0.10 (0.32)	-0.36 (0.49)	NA	NA	0.17 (0.57)	0.12 (0.48)	NA	NA	-0.11 (0.36)	-0.02 (0.32)	-0.41 (1.36)	-0.28 (1.21)
Bare Ground Cover	-0.16 (0.52)	-0.43 (0.68)	NA	NA	-0.24 (0.81)	-0.13 (0.61)	NA	NA	-0.44 (1.45)	-0.29 (1.35)	-0.19 (0.63)	-0.20 (0.64)
Stem Density	-0.38 (1.26)	0.21 (1.21)	NA	NA	0.07 (0.23)	-0.56 (1.43)	NA	NA	-0.23 (0.77)	0.31 (0.84)	-0.20 (0.66)	0.40 (0.97)
R-squared	0.638	0.076	NA	NA	0.131	0.192	NA	NA	0.255	0.080	0.144	0.089
Cumulative R-squared	0.714		NA		0.323		NA		0.335		0.233	

Variable weights (wts.) indicate the direction of the effect and VIP scores > 1 indicate a significant contribution of that variable to the variation explained by each component. Variables explaining a significant amount of the variation in the first component are highlighted in blue (positive) and red (negative). ‘NA’ indicates too few individuals of that species and life stage were collected in the study to perform PLSR.

**Table 4:** PLSR model results for abundance of *I*. *brunneus* and *I*. *spinipalpis*.

Variable	*Ixodes brunneus*						*Ixodes spinipalpis*					
	Adults		Nymphs		Larvae		Adults		Nymphs		Larvae	
	Wts. (VIP) Comp. 1	Wts. (VIP) Comp. 2	Wts. (VIP) Comp. 1	Wts. (VIP) Comp. 2	Wts. (VIP) Comp. 1	Wts. (VIP) Comp. 2	Wts. (VIP) Comp. 1	Wts. (VIP) Comp. 2	Wts. (VIP) Comp. 1	Wts. (VIP) Comp. 2	Wts. (VIP) Comp. 1	Wts. (VIP) Comp. 2
Site	-0.35 (1.17)	0.26 (1.14)	-0.39 (1.30)	0.20 (1.19)	-0.29 (0.96)	0.27 (0.95)	NA	NA	-0.30 (0.98)	0.24 (0.94)	-0.25 (0.83)	-0.06 (0.70)
Avg. Summer Temp.	-0.46 (1.54)	-0.20 (1.47)	-0.59 (1.94)	-0.56 (1.92)	-0.66 (2.20)	-0.42 (2.09)	NA	NA	-0.74 (2.45)	-0.46 (2.28)	-0.77 (2.56)	-0.25 (2.20)
Avg. Winter Temp.	-0.14 (0.47)	0.25 (0.52)	-0.07 (0.23)	-0.01 (0.21)	-0.15 (0.48)	0.29 (0.58)	NA	NA	-0.02 (0.06)	0.38 (0.59)	-0.04 (0.14)	0.73 (1.30)
Elevation	-0.32 (1.07)	0.36 (1.08)	-0.45 (1.48)	0.24 (1.36)	-0.37 (1.21)	0.31 (1.18)	NA	NA	-0.34 (1.12)	0.33 (1.12)	-0.24 (0.79)	0.10 (0.69)
Slope	0.15 (0.49)	0.35 (0.61)	-0.26 (0.86)	0.12 (0.78)	-0.16 (0.53)	-0.01 (0.49)	NA	NA	-0.13 (0.42)	0.11 (0.41)	0.15 (0.50)	0.05 (0.43)
Canopy Cover	0.02 (0.06)	0.64 (0.72)	-0.19 (0.63)	0.36 (0.79)	-0.10 (0.33)	0.29 (0.48)	NA	NA	-0.11 (0.37)	0.26 (0.52)	0.05 (0.16)	0.05 (0.16)
Leaf Litter Cover	0.06 (0.20)	0.28 (0.37)	0.09 (0.29)	0.50 (0.83)	0.19 (0.61)	0.41 (0.77)	NA	NA	0.15 (0.50)	0.33 (0.68)	0.14 (0.46)	-0.14 (0.46)
Shrub Cover	-0.06 (0.20)	-0.27 (0.35)	0.20 (0.65)	-0.28 (0.72)	0.12 (0.41)	-0.47 (0.72)	NA	NA	0.19 (0.63)	-0.44 (0.88)	0.16 (0.53)	-0.36 (0.78)
Herbaceous Cover	0.15 (0.51)	0.13 (0.50)	-0.16 (0.54)	0.04 (0.48)	-0.11 (0.36)	0.25 (0.47)	NA	NA	-0.18 (0.58)	0.24 (0.64)	-0.13 (0.41)	0.37 (0.75)
Bare Ground Cover	-0.66 (2.19)	-0.04 (2.06)	-0.31 (1.03)	-0.08 (0.91)	-0.47 (1.56)	-0.02 (1.44)	NA	NA	-0.36 (1.20)	0.05 (1.06)	-0.45 (1.48)	0.12 (1.26)
Stem Density	-0.22 (0.73)	-0.02 (0.69)	-0.17 (0.56)	0.33 (0.71)	-0.07 (0.22)	0.21 (0.34)	NA	NA	-0.08 (0.26)	0.19 (0.38)	-0.05 (0.16)	-0.31 (0.57)
R-squared	0.235	0.030	0.234	0.068	0.299	0.054	NA	NA	0.307	0.087	0.296	0.121
Cumulative R-squared	0.265		0.302		0.353		NA		0.394		0.417	

Variable weights (wts.) indicate the direction of the effect and VIP scores > 1 indicate a significant contribution of that variable to the variation explained by each component. Variables explaining a significant amount of the variation in the first component are highlighted in blue (positive) and red (negative). ‘NA’ indicates too few individuals of that species and life stage were collected in the study to perform PLSR.

**Table 5:** PLSR model results for abundance of *I*. *peromysci* and tick diversity.

Variable	*Ixodes peromysci*						Tick Diversity (H)	
	Adults		Nymphs		Larvae		All Life Stages	
	Wts. (VIP) Comp. 1	Wts. (VIP) Comp. 2	Wts. (VIP) Comp. 1	Wts. (VIP) Comp. 2	Wts. (VIP) Comp. 1	Wts. (VIP) Comp. 2	Wts. (VIP) Comp. 1	Wts. (VIP) Comp. 2
Site	NA	NA	-0.30 (0.98)	0.24 (0.94)	NA	NA	0.17 (0.56)	-0.51 (1.69)
Avg. Summer Temp.	NA	NA	-0.74 (2.45)	-0.46 (2.28)	NA	NA	-0.26 (0.87)	-0.13 (0.41)
Avg. Winter Temp.	NA	NA	-0.02 (0.06)	0.38 (0.59)	NA	NA	-0.08 (0.27)	0.49 (1.64)
Elevation	NA	NA	-0.34 (1.12)	0.33 (1.12)	NA	NA	0.32 (1.05)	-0.14 (0.47)
Slope	NA	NA	-0.13 (0.42)	0.11 (0.41)	NA	NA	0.28 (0.93)	-0.23 (0.77)
Canopy Cover	NA	NA	-0.11 (0.37)	0.26 (0.52)	NA	NA	0.48 (1.60)	0.11 (0.37)
Leaf Litter Cover	NA	NA	0.15 (0.50)	0.33 (0.68)	NA	NA	0.50 (1.65)	0.20 (0.66)
Shrub Cover	NA	NA	0.19 (0.63)	-0.44 (0.88)	NA	NA	-0.01 (0.02)	0.09 (0.29)
Herbaceous Cover	NA	NA	-0.18 (0.58)	0.24 (0.64)	NA	NA	-0.17 (0.56)	-0.07 (0.25)
Bare Ground Cover	NA	NA	-0.36 (1.20)	0.05 (1.06)	NA	NA	-0.28 (0.92)	-0.51 (1.70)
Stem Density	NA	NA	-0.08 (0.26)	0.19 (0.38)	NA	NA	0.36 (1.20)	-0.28 (0.94)
R-squared	NA	NA	0.307	0.087	NA	NA	0.285	0.154
Cumulative R-squared	NA		0.394		NA		0.439	

Variable weights (wts.) indicate the direction of the effect and VIP scores > 1 indicate a significant contribution of that variable to the variation explained by each component. Variables explaining a significant amount of the variation in the first component are highlighted in blue (positive) and red (negative). ‘NA’ indicates too few individuals of that species and life stage were collected in the study to perform PLSR.

The results of the models predicting abundance of *I*. *pacificus* suggest that dense forest cover and relatively cool and moist microclimates are strong predictors of abundance of all life stages of this species (Table 2). All life stages of *I*. *pacificus* were found to have a strong positive association with elevation, canopy cover and stem density, while the adult stage was found in higher abundance in sites with lower average winter temperatures, and the juvenile stages were additionally found to be strongly positively associated with dense leaf litter microhabitats (Table 2). In contrast, adult *D*. *occidentalis* were positively associated with higher average summer time temperatures, while nymphs of this species were positively associated with higher average winter temperatures, preceding their peak in activity in the summer months (Table 2 and Fig 3), as well as dense leaf litter cover. Larval *D*. *occidentalis* were also strongly associated with dense forest cover (canopy cover, stem density and dense leaf litter cover), but negatively associated with shrub cover (Table 2).

Adult *D*. *variabilis* were found at low elevation and strongly negatively associated with dense forest cover (Table 3). While no nymphal *D*. *variabilis* were collected in this study, the larval stage was found to be strongly associated with dense canopy cover, in contrast with the adult stage (Table 3). Similarly, juvenile *H*. *leporispalustris* were found at lower elevations and were negatively associated with dense forest habitats, instead associated with more open habitats dominated by herbaceous and shrub cover (Table 3). Finally, all life stages of *I*. *brunneus*, as well as juvenile *I*. *spinipalpis* and nymphal *I*. *peromysci*, were all found in lower elevation sites and were strongly negatively associated with higher average summer time temperatures and negatively associated with bare ground microhabitats (Tables 4 and 5). However, these three species (*I*. *brunneus*, *I*. *spinipalpis*, and *I*. *peromysci*) were comparatively rare and models produced poorer overall fits to the data than for other species.

Overall tick diversity, as measured by Shannon’s H-index, which accounts for abundance, richness and evenness of a community, was strongly positively associated with elevation, stem density, canopy cover and dense leaf litter cover (Table 5). In other words, higher levels of tick alpha (local) diversity were supported by dense forest habitats with comparatively cool and moist microclimates.

## Discussion

Many tick-borne diseases have emerged across the northern hemisphere in recent decades, including Lyme disease, Crimean-Congo hemorrhagic fever and tick-borne encephalitis virus. Thus, identifying the abiotic and environmental drivers of tick vector distribution and abundance has become a pressing public health concern. This challenge is all the more salient as processes of global change alter land use and land cover, as well as climate and weather conditions, which will likely alter future patterns of vector distribution and abundance. In this study, the phenology and abiotic and habitat drivers of abundance and diversity of the tick vector community in Santa Barbara County, California were investigated. While there are numerous endemic and emerging tick-borne diseases in California, including Lyme disease, Rocky Mountain Spotted Fever and Pacific Coast Tick Fever, there remains a lack of information about the drivers of the distribution and abundance of the tick vectors, with the exception of *I*. *pacificus*, the primary vector of Lyme disease in the west. This study has contributed to filling this gap.

The primary vectors of public health importance encountered in this study were *I*. *pacificus* (known and likely vector of the causative agents of Lyme disease, anaplasmosis, babesiosis and tick-borne relapsing fever), *D*. *occidentalis* and *D*. *variabilis* (known and likely vectors of Rocky Mountain Spotted Fever, Pacific Coast Tick Fever and Tularemia) which were the most abundant species in this study and, between the three species, encountered year-round. However, each of these species occupied a different niche, both temporally and as defined by abiotic and habitat conditions. For example, *I*. *pacificus*, while abundant throughout the study area, was primarily active in the winter and spring, and constrained to dense forest habitats and cool and moist microclimates. On the other hand, *D*. *occidentalis* was much more tolerant of higher average temperatures and was active later into the summer months, and *D*. *variabilis* was further found in abundance throughout the summer and in more open habitats, in contrast with *I*. *pacificus*. While this suggests year-round risk of encountering medically important tick vectors in California, the landscapes and habitats where risk is highest will shift throughout the year, from dense woodlands in the winter and spring to more open grassland or chaparral habitats in the summer. However, the juvenile stages, which are often more epidemiologically important due to their small size, were much more narrowly distributed and constrained by abiotic conditions across these three species. For example, juveniles of all three species were found to be strongly associated with dense forest microhabitats and displayed a narrower range of seasonal activity than did the adults.

While these species have direct medical relevance to humans, due to their role in zoonotic transmission of pathogens, they comprise a small subset of the tick vector community in California [27]. Yet, ticks of medical importance dominate the literature leaving us with a much more limited understanding of the ecology of the rest of the vector community. This is despite the fact that numerous other tick species are known or suspected to play a role in enzootic pathogen transmission cycles [14,32,33], or have been found to be infected with human pathogens with limited understanding of the possible role they may be playing in maintaining pathogens in natural systems [23]. While these species, like *I*. *spinipalpis* or *H*. *leporispalustris*, do not commonly bite humans, they may play a key role in maintaining human pathogens in natural transmission cycles and thus may in part determine risk of zoonotic transmission and human disease. Interestingly, in this study suspected enzootic vectors of pathogens transmitted to humans by *I*. *pacificus* were similarly restricted in their seasonal activity and abiotic associations. For example, *I*. *spinipalpis* was active only in the winter and spring and was found to be very narrowly distributed and only at low elevation sites and strongly negatively associated with higher average summer temperatures. While these two species shared similar abiotic drivers and patterns of seasonal activity, they exhibited relatively little spatial overlap, with *I*. *pacificus* primarily found at Paradise Reserve and in heavily forested plots in Sedgwick Reserve, and *I*. *spinipalpis* primarily found at Coal Oil Point Reserve. *I*. *spinipalpis* has previously been described as primarily a nidicolous tick displaying endophilic host-seeking behavior [34], so this species may have been more widespread in the study area than the results of drag sampling in this study suggest. Estimates of abundance and seasonal activity of *I*. *brunneus* and *I*. *peromysci* may have been similarly impaired, and resulting measures of overall diversity. *I*. *brunneus*, which primarily parasitizes passerine birds, has been found to quest for hosts on relatively low vegetation or on the ground surface in other regions of the US [35], while relatively little is known about the behavior of *I*. *peromysci*.

While drag sampling is an effective method to determine the relative abundance of actively host-seeking tick species and life stages, it may not be as effective for endophilic species or life stages—those more closely associated with host nests or burrows. As a result, this method is a good proxy for human encounter risk, but may not produce good estimates of abundance of endophilic tick species. This could result in more limited detection of potential enzootic vectors, which may be more host-specific and exhibit endophilic host-seeking behavior. For example, in this study drag sampling appears to underestimate abundance and poorly characterize seasonal activity of the juvenile stages of *D*. *variabilis*, *I*. *spinipalpis* and *I*. *peromysci* relative to host sampling, which may be indicative of differing (possibly more endophilic or nidicolous) host-seeking behavior. Other species (e.g. *I*. *brunneus* and *H*. *leporispalustris*), which have more specialist host-feeding preferences (on birds and lagomorphs respectively), were poorly characterized by small vertebrate host sampling in this study, but appear to be fairly well characterized by drag sampling. These differences in host-seeking behavior and host-feeding preferences may produce unreliable estimates of abundance and seasonal activity patterns. Thus, while broad patterns of geographic distribution (e.g. distribution across the three reserves) as determined by drag and host sampling techniques mirror each other, model results of specific habitat and abiotic drivers of these tick species and life stages should be regarded with some caution. More targeted studies of these species and life stages are warranted before drawing conclusions about these drivers and how abundance and distribution of these species might be expected to shift under projected environmental change.

Nonetheless, some species thought to be involved in enzootic transmission cycles for the causative agents of Lyme disease [20,34] and Rocky Mountain Spotted Fever [36] have been found to exhibit more active, or exophilic, host-seeking behavior elsewhere in the United States. These species have previously been successfully collected, though some with low capture rates, in Santa Barbara County using the drag method [23]. Future studies using sentinel animals to determine seasonal activity and distribution in southern California of *I*. *spinipalpis* and other potentially important enzootic vectors are warranted. Such studies may uncover additional host-specialist species in this understudied region that would not have been detected by the drag method in this study. Where there was evidence of life stage specific host-seeking behavior (e.g. low capture rate of juvenile *D*. *variabilis* relative to adults via drag sampling) from this study, future work would benefit from an experimental investigation of host-seeking behavior in addition to concurrent host sampling to determine the degree to which sampling method may impact estimates of abundance and seasonal activity. Such an approach would also aid in determining the extent to which changing vegetation structure, and resulting microclimates for ticks, over the course of the year might influence estimates of abundance via drag sampling.

In contrast, *H*. *leporispalustris*, which displays exophilic host-seeking behavior [36] and is a suspected enzootic vector of pathogens transmitted to humans by *Dermacentor* species ticks, was widespread throughout the study area, active throughout the spring and into the summer and associated with more open habitats. This spatial and temporal overlap in activity and abundance of *H*. *leporispalustris* and *Dermacentor* species ticks was expected and may promote zoonotic transmission of *Dermacentor* transmitted pathogens [22]. The limited overlap between *I*. *pacificus* and potential enzootic vectors (e.g. *I*. *spinipalpis*), and substantial overlap between *Dermacentor* species and *H*. *leporispalustris* found in this study suggest that there may be more interaction between enzootic transmission cycles and zoonotic vectors for *Dermacentor* transmitted pathogens than for *I*. *pacificus* transmitted pathogens. However, this deserves further investigation, particularly in assessing the degree to which enzootic transmission of pathogens occurs in this region, as pathogen screening was not undertaken in this study. Due to the possible endophilic nature of the enzootic vector species, such a study would benefit from tracking small vertebrate hosts and screening them for pathogens along with collected ticks.

The results of this study further suggest that changing land cover and climate are likely to have differential impacts on the distribution, abundance and seasonal activity of tick vectors involved in zoonotic and enzootic transmission of human pathogens in this region of California. In California, climate change projections suggest an overall trend toward increasing temperatures, particularly in the summer months, declines in precipitation, and elevated drought risk [37–39]. With more arid climates and changing fire regimes [40], land cover will be expected to shift as well, likely supporting less and more sparse forest habitat [41]. These changes are likely to have different effects on the distribution, abundance and seasonal activity of tick vectors across southern California. For example, while some regions of northwestern California may become more suitable that are currently too seasonally cold to support robust populations of *I*. *pacificus*, it is likely that many other regions (e.g. southern or inland regions of California) will become too hot and dry, particularly in summer months, for high inter-annual survivorship of *I*. *pacificus* [11]. These same regions are likely to experience reductions in seasonal activity, or shifts in when peak activity occurs to earlier in the winter months [9]. Additionally, vector diversity, associated with dense woodland and cool and moist microclimates in this study as well as with elevated infection prevalence with *B*. *burgdorferi* sensu lato in an earlier study in this region [23], may also be expected to decline regionally into the future.

While *I*. *pacificus* may be expected to experience overall reductions in suitable habitat and microclimates, as well as more limited seasonal activity, other tick species may fare much better in California’s changing climates and landscapes. For example, *D*. *occidentalis*, which in this study was associated with warmer average temperatures, and *D*. *variabilis*, which was associated with more open habitats, may be expected to expand in geographic distribution, and experience more limited effects of climate warming on overall abundance and seasonal activity. *H*. *leporispalustris*, a suspected enzootic vector of *Rickettsia rickettsii*, was found to be widely distributed throughout the study area and more tolerant of open habitats and higher temperatures, which suggests it may too experience more limited effects of climate warming and land cover change.

These divergent predictions suggest that tick-borne disease risk may also shift regionally in the west with pathogens transmitted by *Dermacentor* or other species of tick (e.g. *Rhipicephalus sanguineus*, for example [42]) becoming more common, and *I*. *pacificus* transmitted pathogens becoming comparably less common. This possible scenario contrasts with predictions in eastern North America where *Ixodes scapularis*, the primary vector of *B*. *burgdorferi* (Lyme disease), *A*. *phagocytophilum* (anaplasmosis) and *Babesia microti* (babesiosis), appears to be expanding in geographic distribution [43–46]. To investigate these predictions, as well as possible shifts in the distribution, abundance and seasonal activity of vector ticks in southern California and the western US broadly would benefit from species distribution modeling approaches, which could capture possible changes over large geographic areas resulting from changing climate and land cover [19]. The data collected for this study may aid such modeling efforts, particularly given the limited availability of vector data in central and southern California. Such large-scale analyses are necessary, particularly where there is a lack of resources available for large field sampling efforts, to predict where important vector species are likely to occur. Such studies will be necessary to predict fine-resolution future distributions of important vector species and diversity of the tick vector community across the western US under projected climate and land cover change, informing changing disease risk into the future. The clear differences observed between vector species in this study suggest that global change will not have uniform outcomes for vector-borne disease risk. Responses to climate and land cover change will be specific to vector species, pathogens and host communities, and may further differ between regions for individual species. Thus, to understand aggregate changes in vector-borne disease risk requires a focus on the vector community, including both enzootic and zoonotic transmission cycles. Such research will better inform vector-borne disease risk, now and in the future.

## Supporting information

S1 Fig Distribution of Mediterranean climates in California. Distribution of Mediterranean climates based on the Koppen-Geiger climate classification; data available at: https://webmap.ornl.gov/ogc/dataset.jsp?ds_id=10012.
(PNG)

S2 Fig Distribution of sampled habitats in California. Distribution of grassland, shrubland and oak/mixed-oak woodland habitats in California. Data is from the California Fire Resource and Assessment Program (FRAP) and represents the “best available” land cover data for the state of California; data available at: http://frap.fire.ca.gov/data/frapgisdata-sw-fveg_download.
(PNG)

S3 Fig Seasonal activity of *I*. *spinipalpis*. Represented as density of ticks per 100m^2^ by month. Adults are in the top panel, nymphs in the middle and larvae on the bottom. Individuals of this species were rarely encountered, and no clear seasonal trends in activity were identified. The three sites, *Coal Oil Point* (white), *Paradise* (light grey) and *Sedgwick* (dark grey), are represented by individual bars in each month, in that order. Black dots represent outliers; horizontal bars in box plots represent the mean; horizontal bars without box plots represent a single sample in which that species/life stage was collected in a given month.
(TIFF)

S4 Fig Seasonal activity of *I*. *peromysci*. Represented as density of ticks per 100m^2^ by month. Adults are in the top panel, nymphs in the middle and larvae on the bottom. Individuals of this species were rarely encountered, and no clear seasonal trends in activity were identified. The three sites, *Coal Oil Point* (white), *Paradise* (light grey) and *Sedgwick* (dark grey), are represented by individual bars in each month, in that order. Black dots represent outliers; horizontal bars in box plots represent the mean; horizontal bars without box plots represent a single sample in which that species/life stage was collected in a given month.
(TIFF)

S5 Fig Seasonal host feeding of *I*. *pacificus*. Represented as density of ticks per host by month. Adults are in the top panel, nymphs in the middle and larvae on the bottom. The three sites, *Coal Oil Point* (white), *Paradise* (light grey) and *Sedgwick* (dark grey), are represented by individual bars in each month, in that order. Black dots represent outliers; horizontal bars in box plots represent the mean; horizontal bars without box plots represent a single sample in which that species/life stage was collected in a given month. Few adults were collected from hosts, because adult *I*. *pacificus* primarily feed on large vertebrate hosts like deer. Geographic distribution and seasonal patterns on hosts largely mirrors what was observed in drag sampling.
(TIFF)

S6 Fig Seasonal host feeding of *D*. *occidentalis*. Represented as density of ticks per host by month. Adults are in the top panel, nymphs in the middle and larvae on the bottom. The three sites, *Coal Oil Point* (white), *Paradise* (light grey) and *Sedgwick* (dark grey), are represented by individual bars in each month, in that order. Black dots represent outliers; horizontal bars in box plots represent the mean; horizontal bars without box plots represent a single sample in which that species/life stage was collected in a given month. Geographic distribution and seasonal patterns on hosts largely mirrors what was observed in drag sampling, though sampling from hosts appears to underestimate abundance relative to drag sampling.
(TIFF)

S7 Fig Seasonal host feeding of *D*. *variabilis*. Represented as density of ticks per host by month. Adults are in the top panel, nymphs in the middle and larvae on the bottom. The three sites, *Coal Oil Point* (white), *Paradise* (light grey) and *Sedgwick* (dark grey), are represented by individual bars in each month, in that order. Black dots represent outliers; horizontal bars in box plots represent the mean; horizontal bars without box plots represent a single sample in which that species/life stage was collected in a given month. Geographic distribution on hosts largely mirrors what was observed in drag sampling (e.g. primarily distributed in Coal Oil Point Reserve), though drag sampling appears to underestimate abundance of juvenile ticks relative to sampling from hosts, which may indicate differing host-seeking behavior.
(TIFF)

S8 Fig Seasonal host feeding of *I*. *spinipalpis*. Represented as density of ticks per host by month. Adults are in the top panel, nymphs in the middle and larvae on the bottom. The three sites, *Coal Oil Point* (white), *Paradise* (light grey) and *Sedgwick* (dark grey), are represented by individual bars in each month, in that order. Black dots represent outliers; horizontal bars in box plots represent the mean; horizontal bars without box plots represent a single sample in which that species/life stage was collected in a given month. Geographic distribution on hosts largely mirrors what was observed in drag sampling (e.g. primarily distributed in Coal Oil Point Reserve), though drag sampling appears to underestimate abundance of juvenile ticks relative to sampling from hosts, which may indicate differing host-seeking behavior.
(TIFF)

S9 Fig Seasonal host feeding of *I*. *brunneus*. Represented as density of ticks per host by month. Adults are in the top panel, nymphs in the middle and larvae on the bottom. The three sites, *Coal Oil Point* (white), *Paradise* (light grey) and *Sedgwick* (dark grey), are represented by individual bars in each month, in that order. Black dots represent outliers; horizontal bars in box plots represent the mean; horizontal bars without box plots represent a single sample in which that species/life stage was collected in a given month. Geographic distribution on hosts largely mirrors what was observed in drag sampling (e.g. primarily distributed in Coal Oil Point Reserve), though sampling from hosts appears to underestimate abundance of ticks relative to drag sampling. This is likely because *I*. *brunneus* primarily parasitizes birds, which were not represented in the hosts sampled.
(TIFF)

S10 Fig Seasonal host feeding of *I*. *peromysci*. Represented as density of ticks per host by month. Adults are in the top panel, nymphs in the middle and larvae on the bottom. The three sites, *Coal Oil Point* (white), *Paradise* (light grey) and *Sedgwick* (dark grey), are represented by individual bars in each month, in that order. Black dots represent outliers; horizontal bars in box plots represent the mean; horizontal bars without box plots represent a single sample in which that species/life stage was collected in a given month. Geographic distribution on hosts largely mirrors what was observed in drag sampling (e.g. primarily distributed in Coal Oil Point Reserve), though drag sampling appears to underestimate abundance of ticks of all life stages relative to sampling from hosts, which may indicate differing host-seeking behavior.
(TIFF)

S11 Fig Seasonal host feeding of *H*. *leporispalustris*. No *H*. *leporispalustris* were encountered on the hosts sampled in this study. This is likely because *H*. *leporispalustris* primarily parasitizes lagomorphs, which were not represented in the hosts sampled.
(TIFF)

S12 Fig Relative abundance of tick species by life stage and reserve as determined by host sampling and drag sampling. First column illustrates relative abundance of tick species by life stage in each reserve as determined by host sampling; second column illustrates relative abundance of tick species by life stage in each reserve as determined by drag sampling. First row illustrates the difference in relative abundance estimates between host and drag sampling at Coal Oil Point (second row = Paradise Reserve; third row = Sedgwick Reserve). Tick species: “DO” = *D*. *occidentalis*, “DV” = *D*. *variabilis*, “Hl” = *H*. *leporispalustris*, “Ib” = *I*. *brunneus*, “IP” = *I*. *pacificus*, “Ipero” = *I*. *peromysci*, and “Is” = *I*. *spinipalpis*. Some species (e.g. “IP” and “DO”) are well characterized by drag sampling, while others (e.g. “Ipero”, “Is” and “DV”) are not.
(TIFF)

S1 Table Number of hosts and host species sampled in each reserve. Host sampling took place from winter of 2013 through spring of 2016 at Coal Oil Point Reserve; in winter, spring and summer of 2014 at Paradise Reserve [24]; and from winter of 2013 through winter of 2015 at Sedgwick Reserve.
(DOCX)
