# Supplementary figures and images for: Abiotic and habitat drivers of tick vector abundance, diversity, phenology and human encounter risk in southern California
Source: PLoS One. 2018 Jul 31;13(7):e0201665. doi: 10.1371/journal.pone.0201665 (PMC6067749; doi:10.1371/journal.pone.0201665)

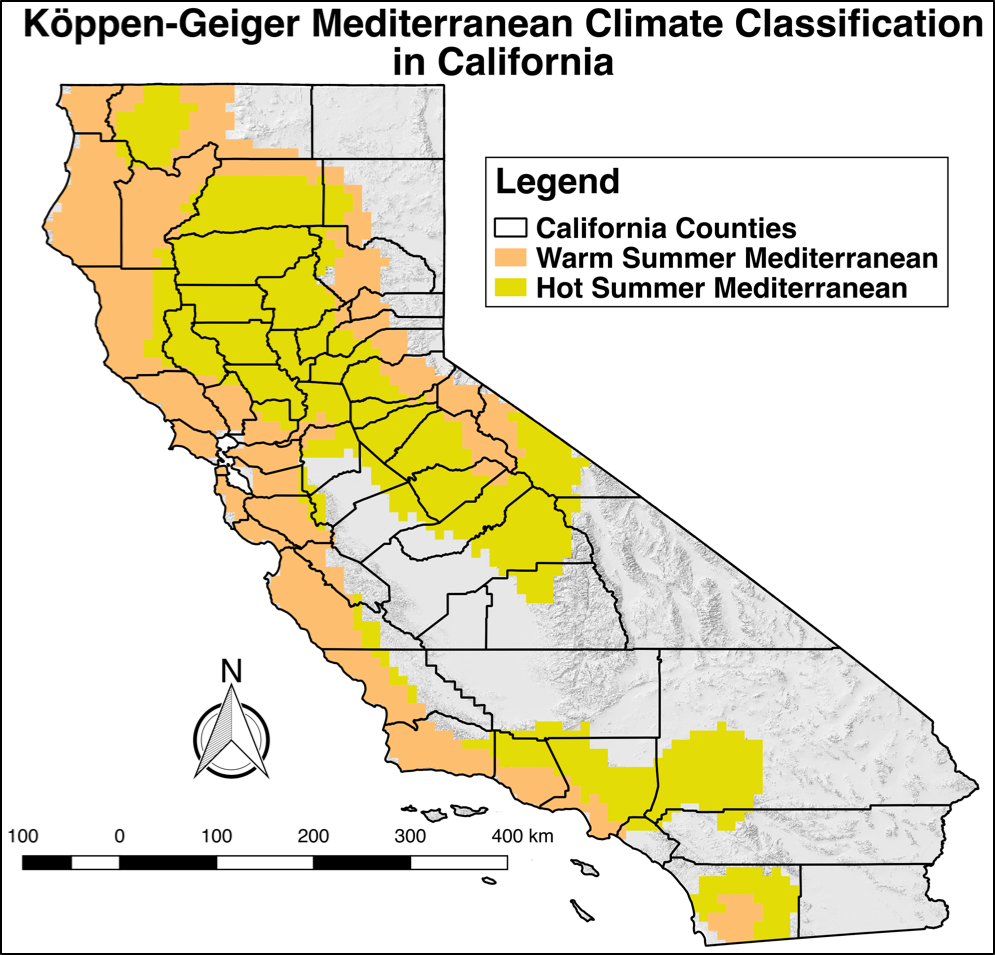

Supplement: S1 Fig — Distribution of Mediterranean climates based on the Koppen-Geiger climate classification; data available at: https://webmap.ornl.gov/ogc/dataset.jsp?ds_id=10012. (PNG) [file pone.0201665.s001.png]

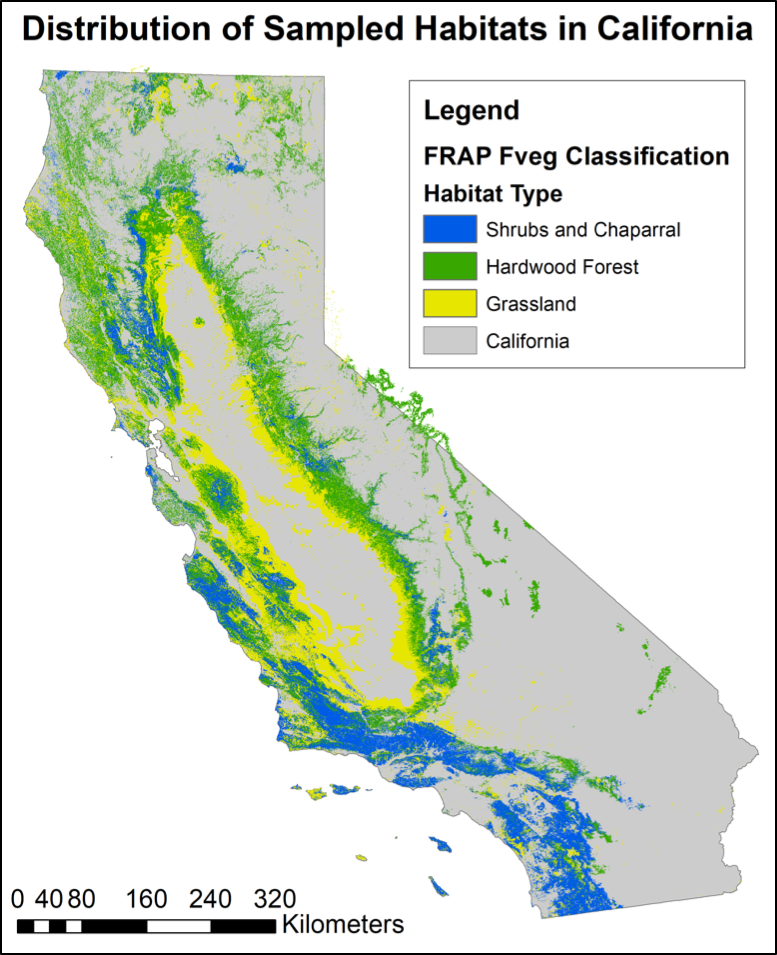

Supplement: S2 Fig — Distribution of grassland, shrubland and oak/mixed-oak woodland habitats in California. Data is from the California Fire Resource and Assessment Program (FRAP) and represents the “best available” land cover data for the state of California; data available at: http://frap.fire.ca.gov/data/frapgisdata-sw-fveg_download. (PNG) [file pone.0201665.s002.png]

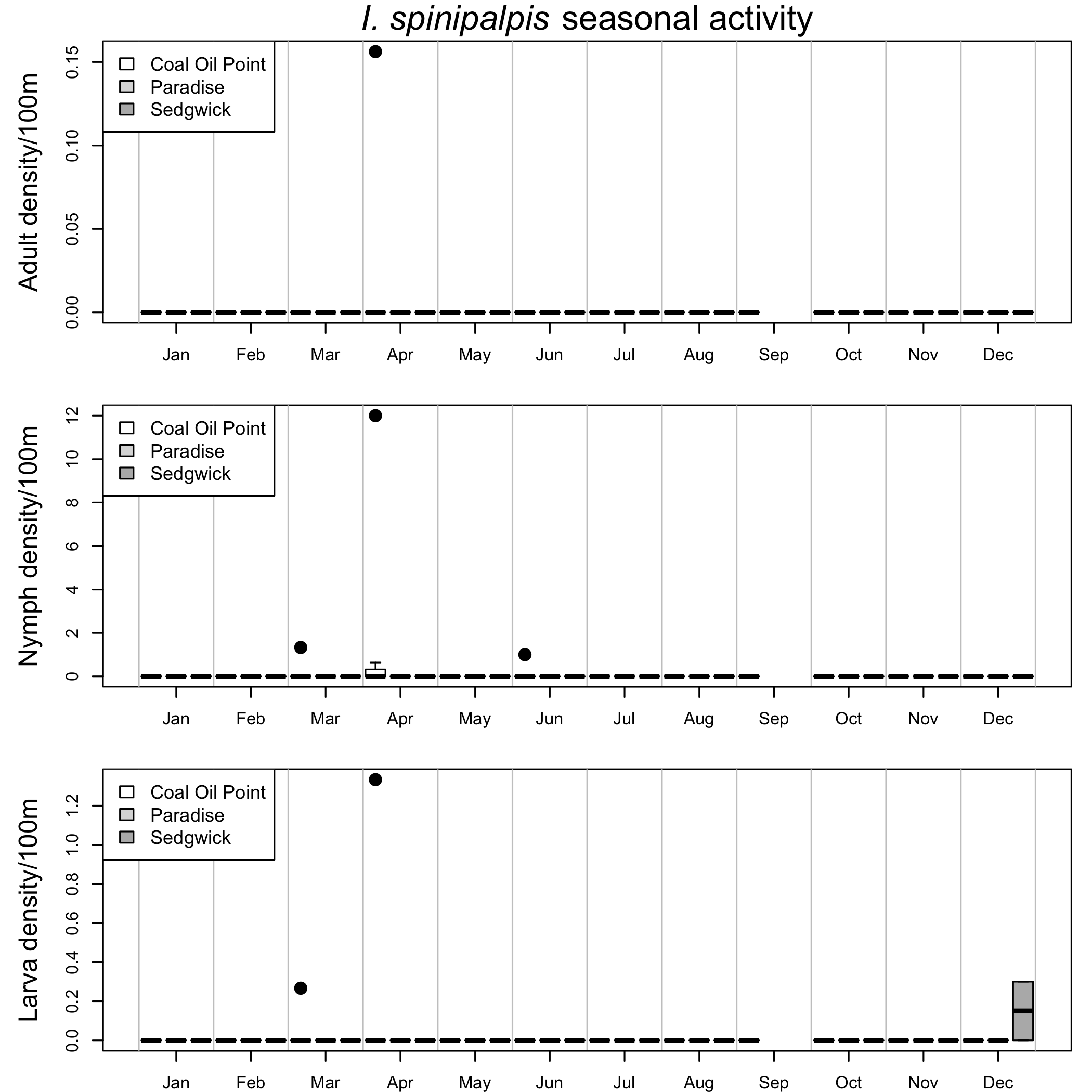

Supplement: S3 Fig — Represented as density of ticks per 100m2 by month. Adults are in the top panel, nymphs in the middle and larvae on the bottom. Individuals of this species were rarely encountered, and no clear seasonal trends in activity were identified. The three sites, Coal Oil Point (white), Paradise (light grey) and Sedgwick (dark grey), are represented by individual bars in each month, in that order. Black dots represent outliers; horizontal bars in box plots represent the mean; horizontal bars without box plots represent a single sample in which that species/life stage was collected in a given month. (TIFF) [file pone.0201665.s003.tiff]

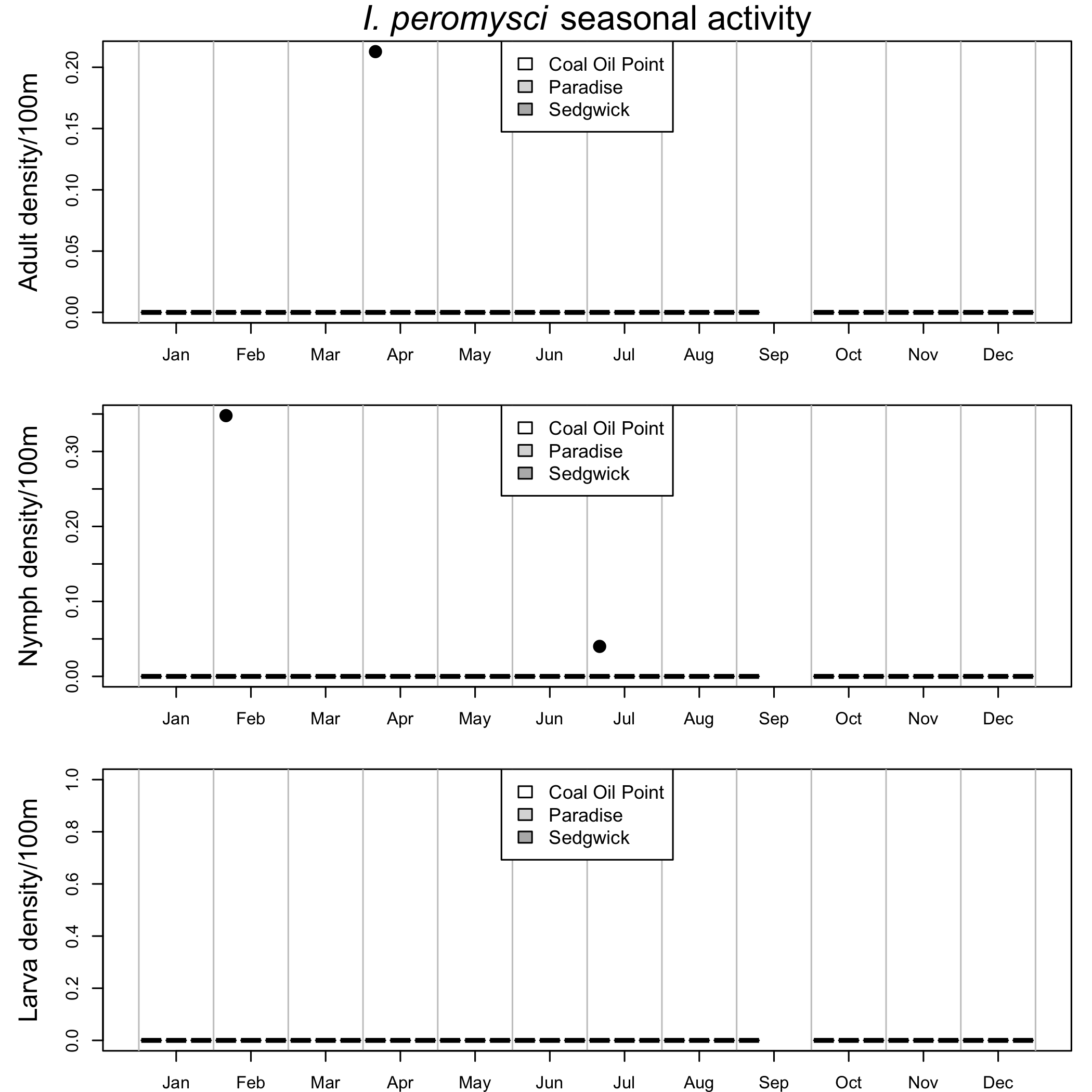

Supplement: S4 Fig — Represented as density of ticks per 100m2 by month. Adults are in the top panel, nymphs in the middle and larvae on the bottom. Individuals of this species were rarely encountered, and no clear seasonal trends in activity were identified. The three sites, Coal Oil Point (white), Paradise (light grey) and Sedgwick (dark grey), are represented by individual bars in each month, in that order. Black dots represent outliers; horizontal bars in box plots represent the mean; horizontal bars without box plots represent a single sample in which that species/life stage was collected in a given month. (TIFF) [file pone.0201665.s004.tiff]

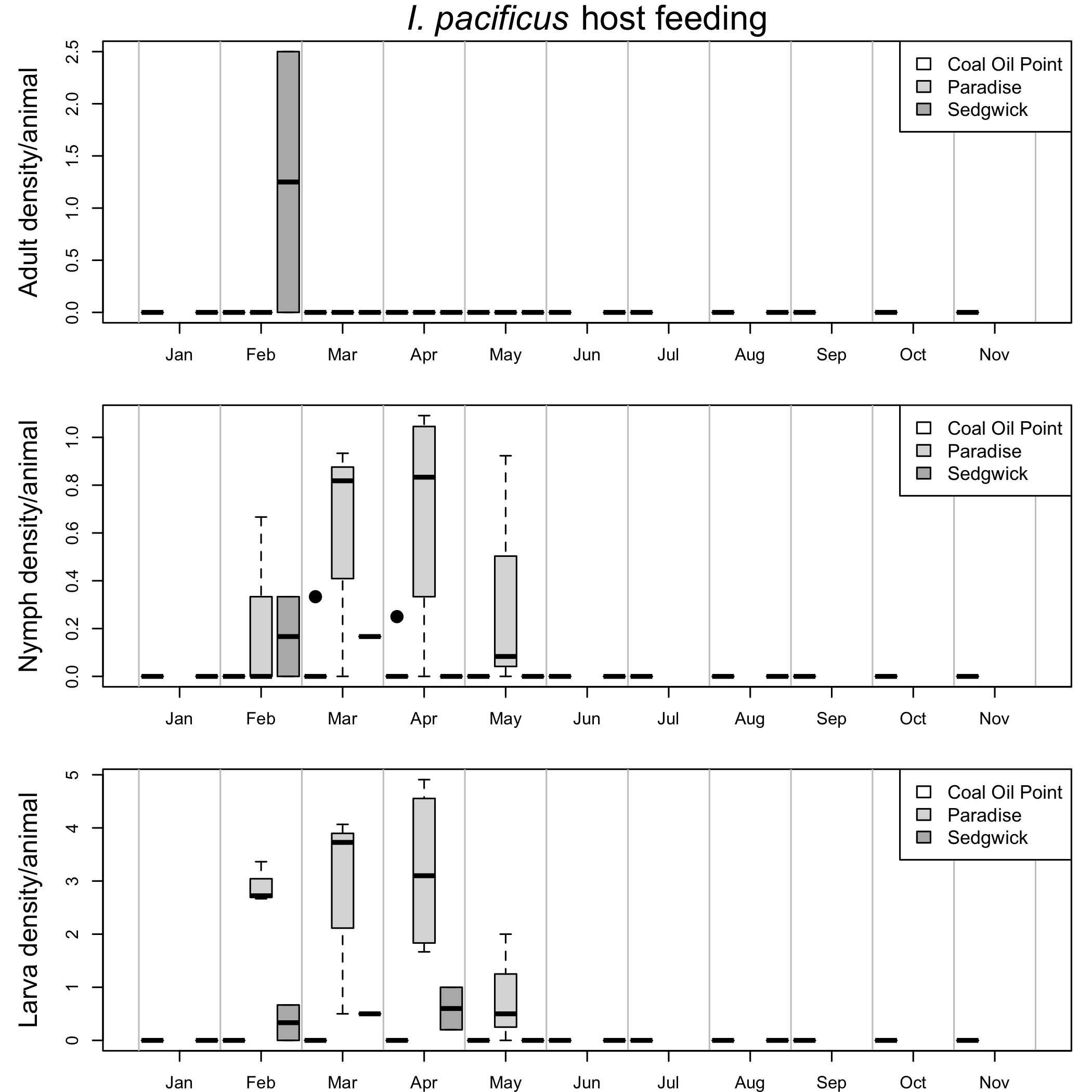

Supplement: S5 Fig — Represented as density of ticks per host by month. Adults are in the top panel, nymphs in the middle and larvae on the bottom. The three sites, Coal Oil Point (white), Paradise (light grey) and Sedgwick (dark grey), are represented by individual bars in each month, in that order. Black dots represent outliers; horizontal bars in box plots represent the mean; horizontal bars without box plots represent a single sample in which that species/life stage was collected in a given month. Few adults were collected from hosts, because adult I. pacificus primarily feed on large vertebrate hosts like deer. Geographic distribution and seasonal patterns on hosts largely mirrors what was observed in drag sampling. (TIFF) [file pone.0201665.s005.tiff]

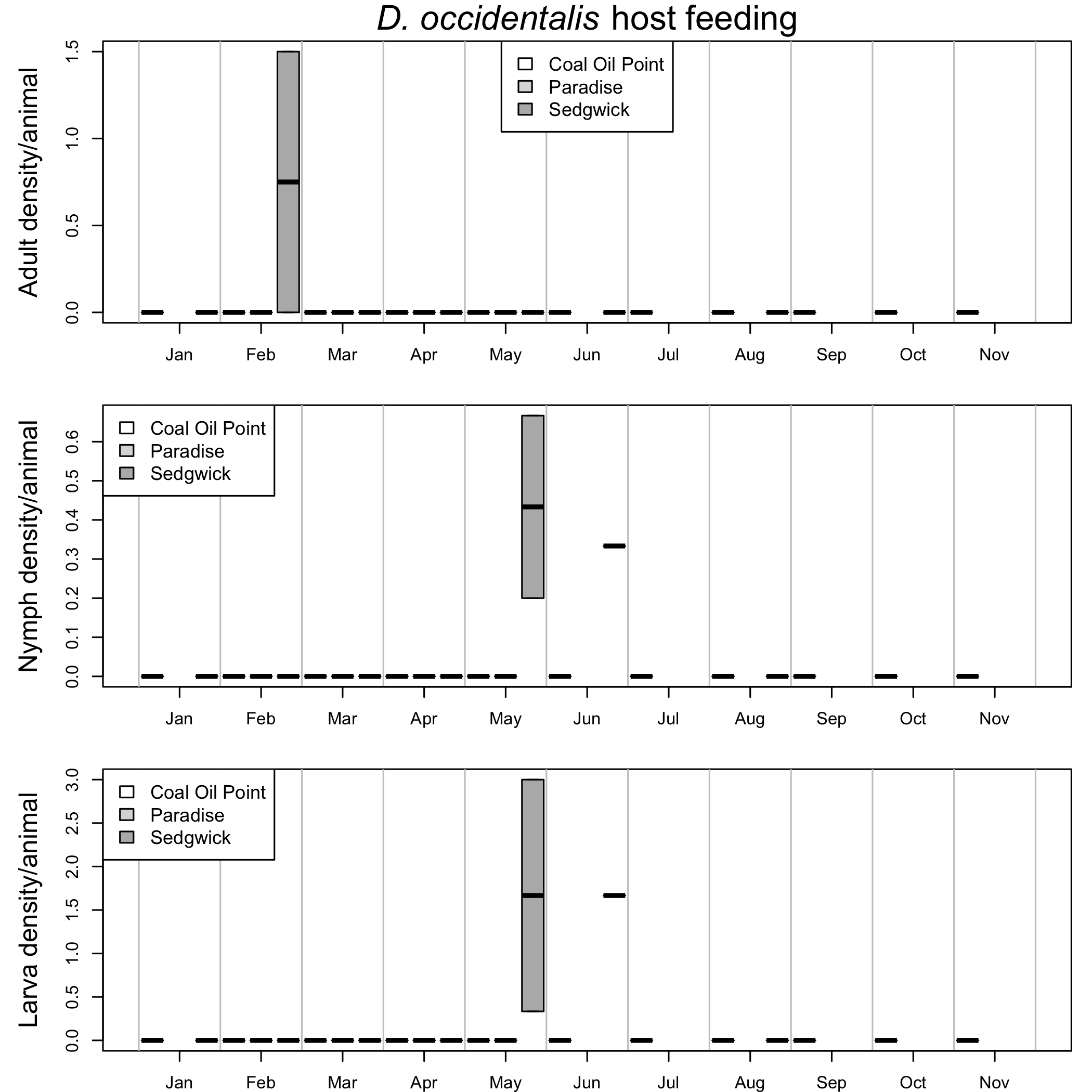

Supplement: S6 Fig — Represented as density of ticks per host by month. Adults are in the top panel, nymphs in the middle and larvae on the bottom. The three sites, Coal Oil Point (white), Paradise (light grey) and Sedgwick (dark grey), are represented by individual bars in each month, in that order. Black dots represent outliers; horizontal bars in box plots represent the mean; horizontal bars without box plots represent a single sample in which that species/life stage was collected in a given month. Geographic distribution and seasonal patterns on hosts largely mirrors what was observed in drag sampling, though sampling from hosts appears to underestimate abundance relative to drag sampling. (TIFF) [file pone.0201665.s006.tiff]

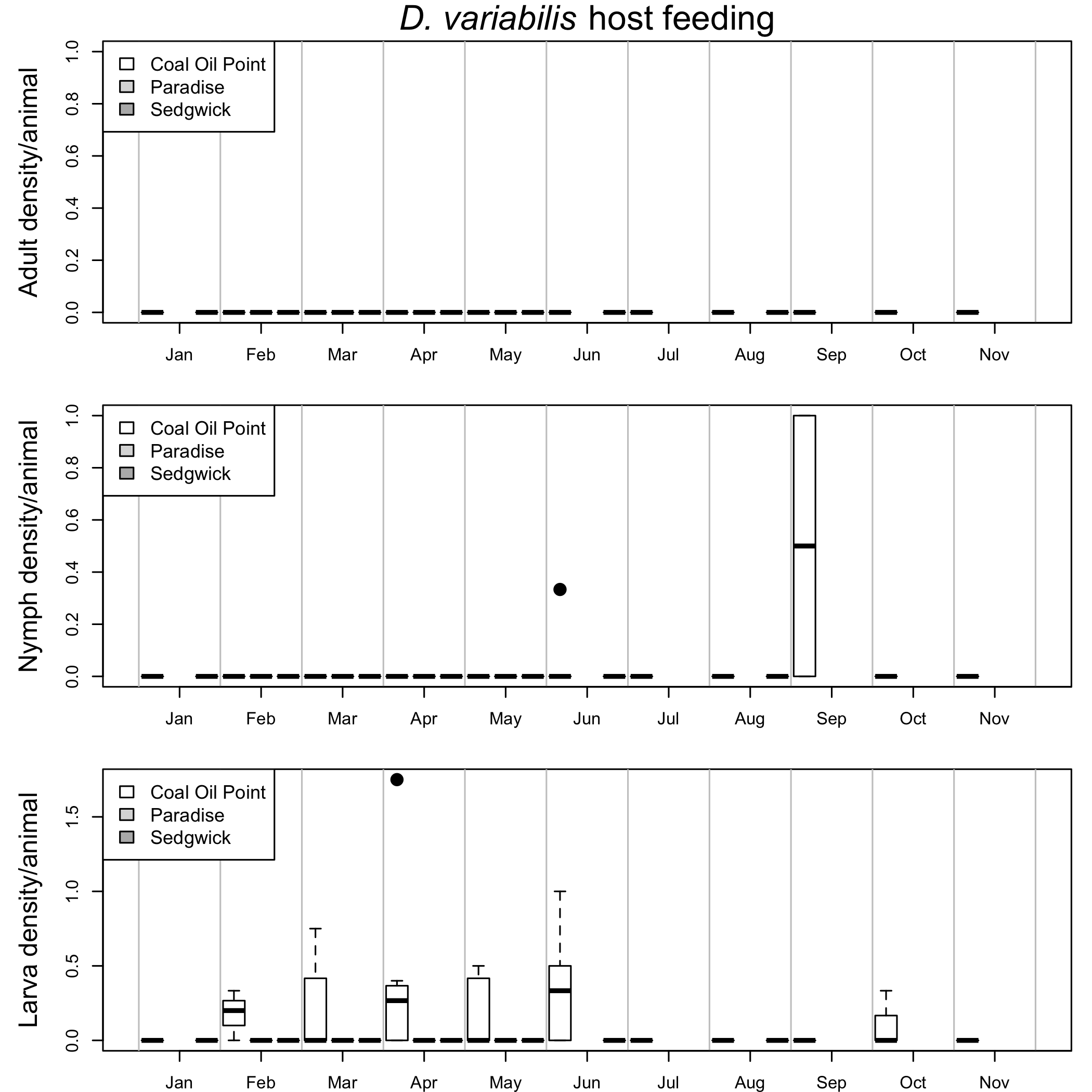

Supplement: S7 Fig — Represented as density of ticks per host by month. Adults are in the top panel, nymphs in the middle and larvae on the bottom. The three sites, Coal Oil Point (white), Paradise (light grey) and Sedgwick (dark grey), are represented by individual bars in each month, in that order. Black dots represent outliers; horizontal bars in box plots represent the mean; horizontal bars without box plots represent a single sample in which that species/life stage was collected in a given month. Geographic distribution on hosts largely mirrors what was observed in drag sampling (e.g. primarily distributed in Coal Oil Point Reserve), though drag sampling appears to underestimate abundance of juvenile ticks relative to sampling from hosts, which may indicate differing host-seeking behavior. (TIFF) [file pone.0201665.s007.tiff]

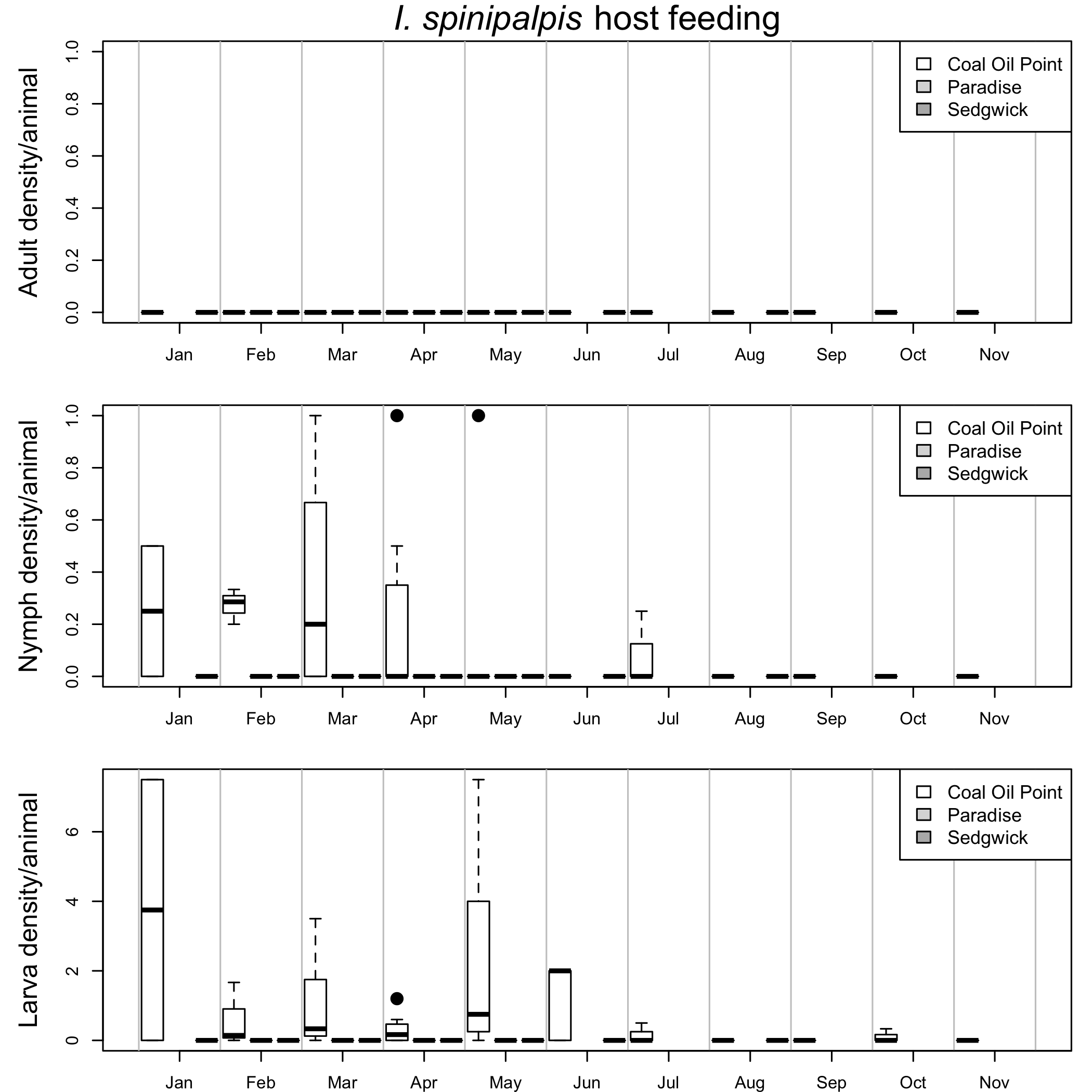

Supplement: S8 Fig — Represented as density of ticks per host by month. Adults are in the top panel, nymphs in the middle and larvae on the bottom. The three sites, Coal Oil Point (white), Paradise (light grey) and Sedgwick (dark grey), are represented by individual bars in each month, in that order. Black dots represent outliers; horizontal bars in box plots represent the mean; horizontal bars without box plots represent a single sample in which that species/life stage was collected in a given month. Geographic distribution on hosts largely mirrors what was observed in drag sampling (e.g. primarily distributed in Coal Oil Point Reserve), though drag sampling appears to underestimate abundance of juvenile ticks relative to sampling from hosts, which may indicate differing host-seeking behavior. (TIFF) [file pone.0201665.s008.tiff]

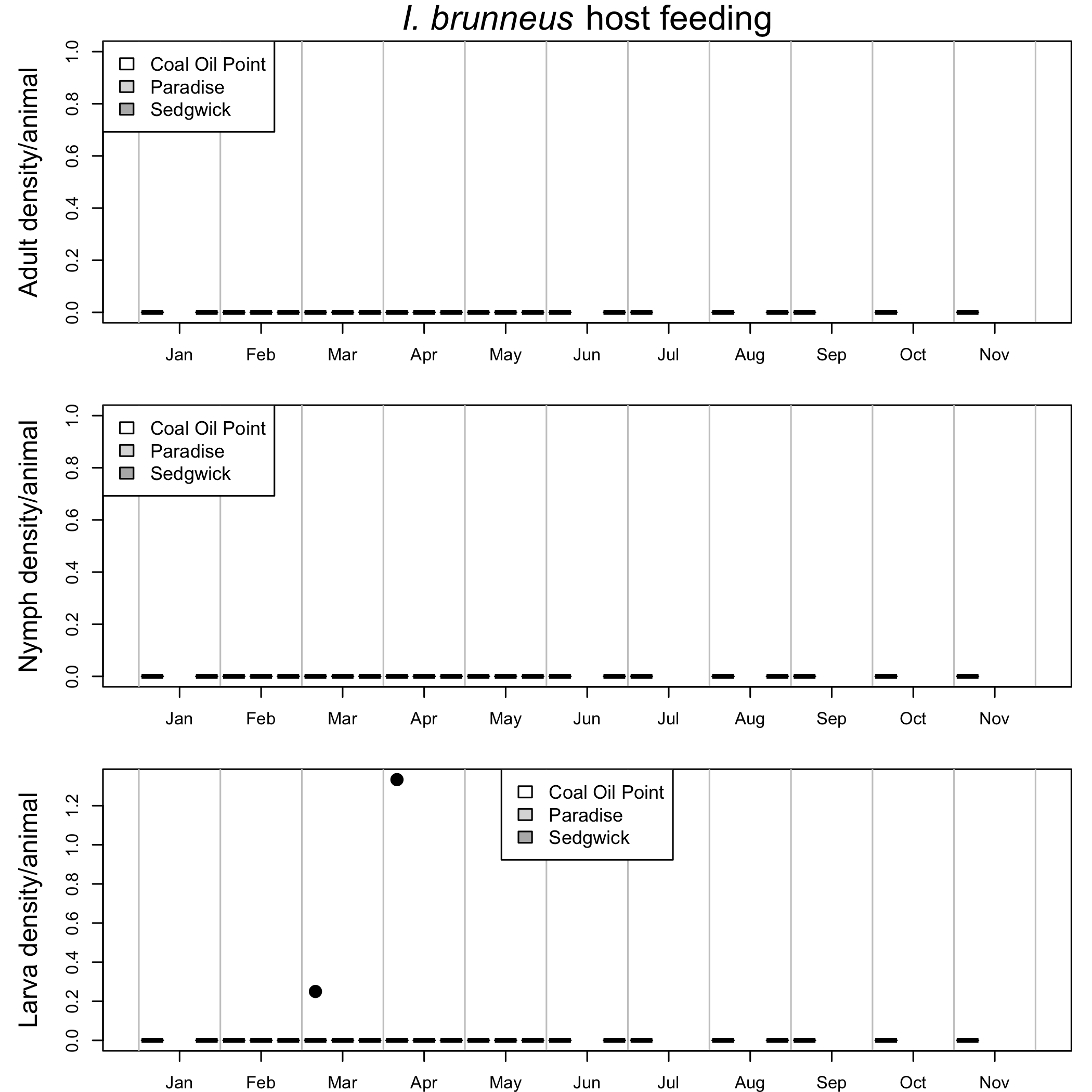

Supplement: S9 Fig — Represented as density of ticks per host by month. Adults are in the top panel, nymphs in the middle and larvae on the bottom. The three sites, Coal Oil Point (white), Paradise (light grey) and Sedgwick (dark grey), are represented by individual bars in each month, in that order. Black dots represent outliers; horizontal bars in box plots represent the mean; horizontal bars without box plots represent a single sample in which that species/life stage was collected in a given month. Geographic distribution on hosts largely mirrors what was observed in drag sampling (e.g. primarily distributed in Coal Oil Point Reserve), though sampling from hosts appears to underestimate abundance of ticks relative to drag sampling. This is likely because I. brunneus primarily parasitizes birds, which were not represented in the hosts sampled. (TIFF) [file pone.0201665.s009.tiff]

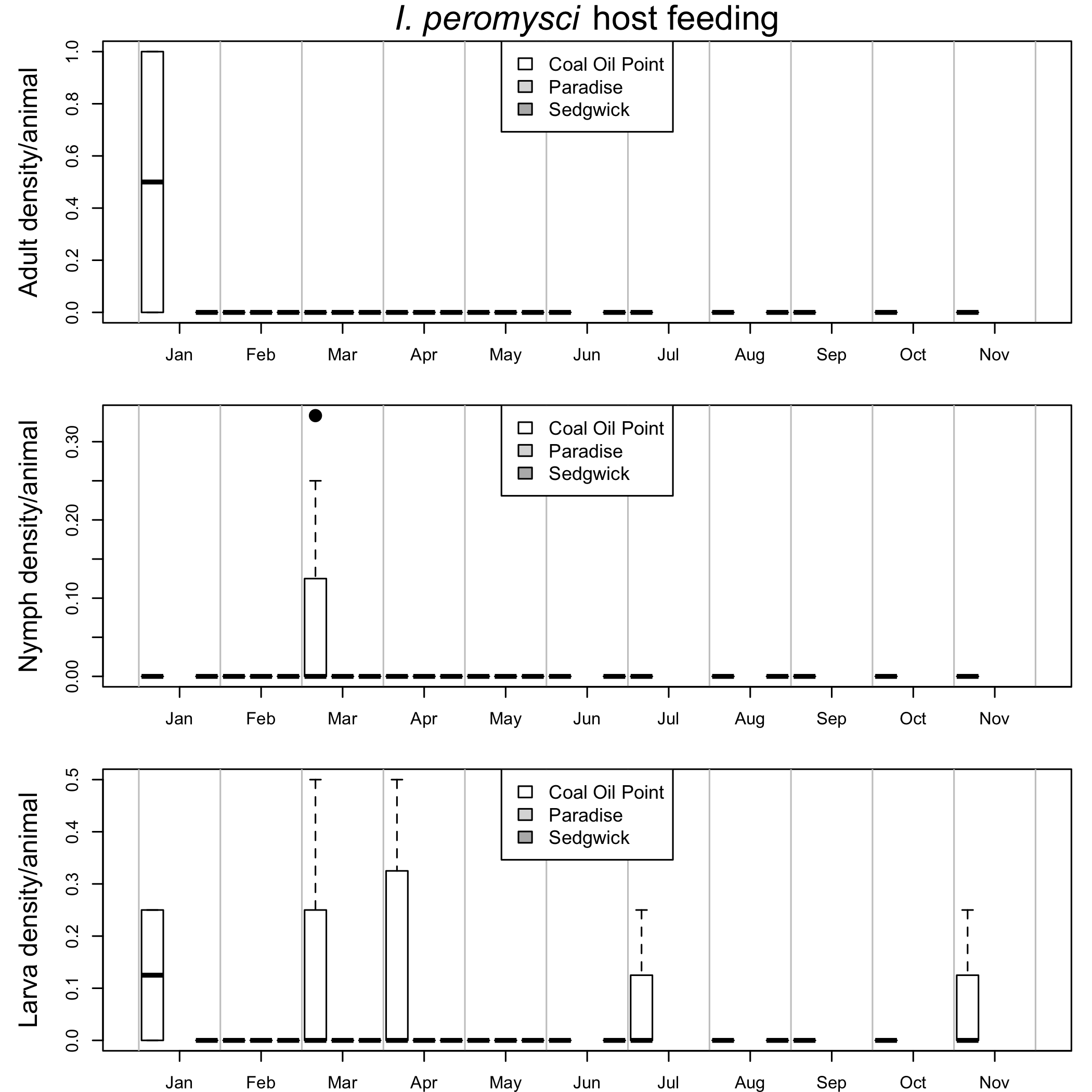

Supplement: S10 Fig — Represented as density of ticks per host by month. Adults are in the top panel, nymphs in the middle and larvae on the bottom. The three sites, Coal Oil Point (white), Paradise (light grey) and Sedgwick (dark grey), are represented by individual bars in each month, in that order. Black dots represent outliers; horizontal bars in box plots represent the mean; horizontal bars without box plots represent a single sample in which that species/life stage was collected in a given month. Geographic distribution on hosts largely mirrors what was observed in drag sampling (e.g. primarily distributed in Coal Oil Point Reserve), though drag sampling appears to underestimate abundance of ticks of all life stages relative to sampling from hosts, which may indicate differing host-seeking behavior. (TIFF) [file pone.0201665.s010.tiff]

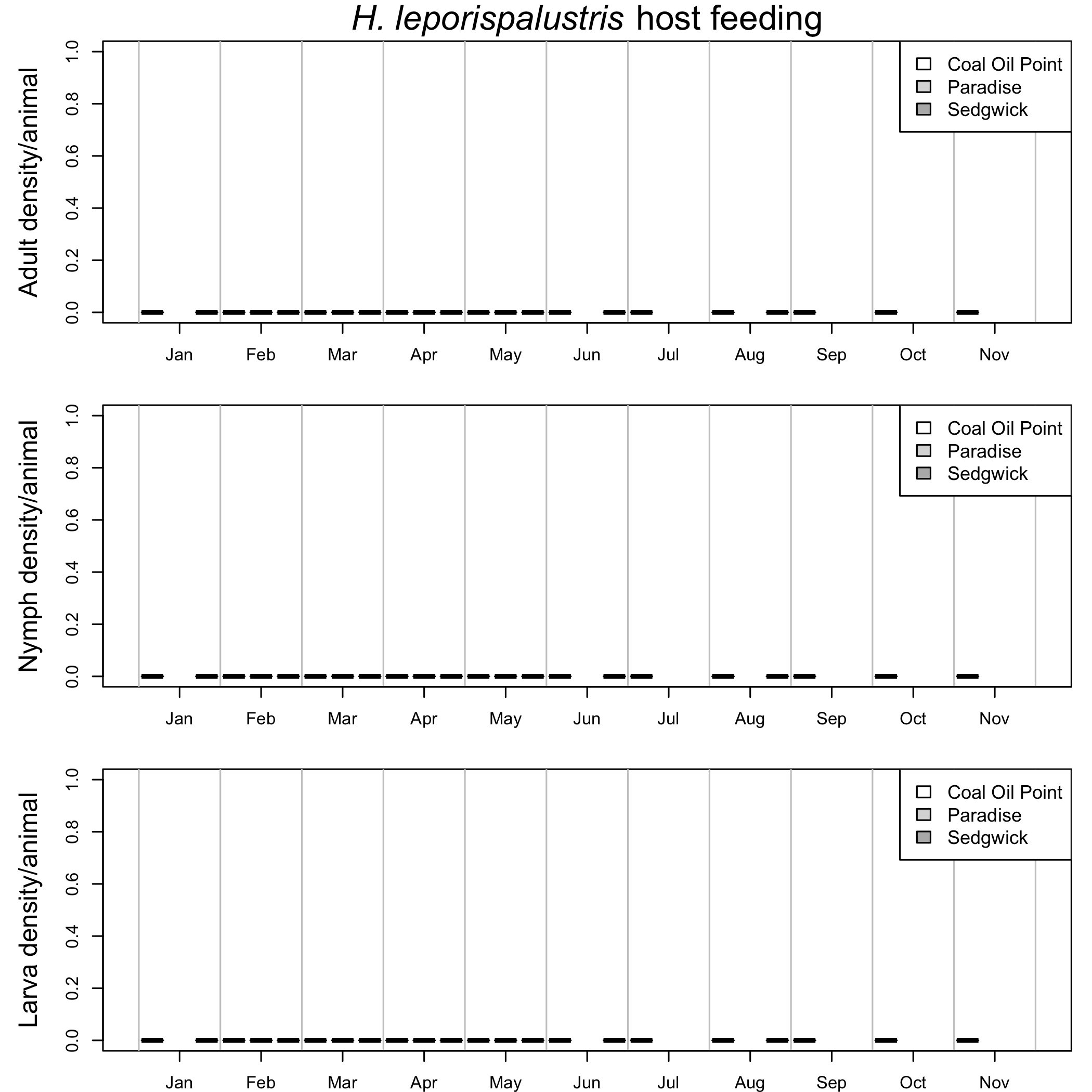

Supplement: S11 Fig — No H. leporispalustris were encountered on the hosts sampled in this study. This is likely because H. leporispalustris primarily parasitizes lagomorphs, which were not represented in the hosts sampled. (TIFF) [file pone.0201665.s011.tiff]

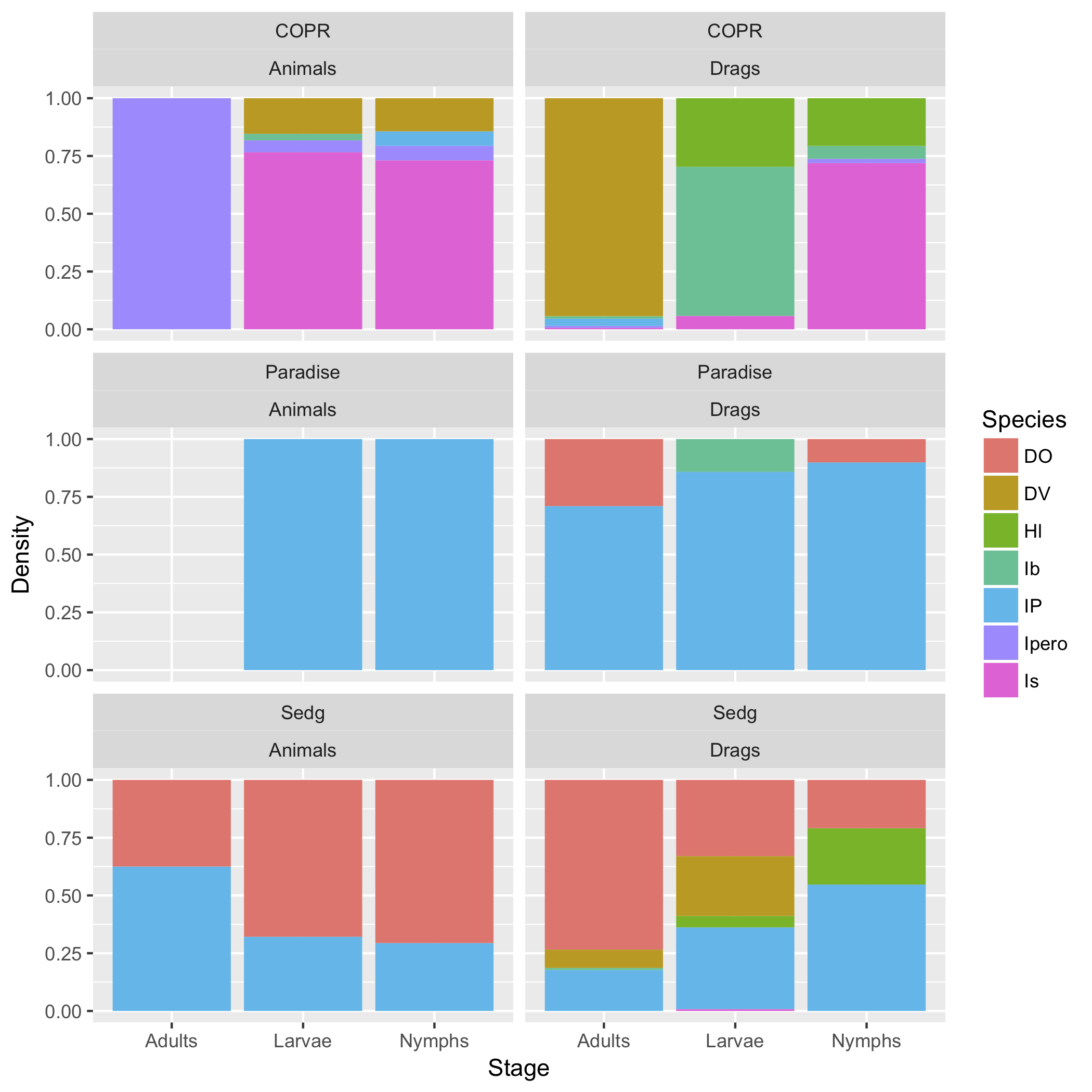

Supplement: S12 Fig — First column illustrates relative abundance of tick species by life stage in each reserve as determined by host sampling; second column illustrates relative abundance of tick species by life stage in each reserve as determined by drag sampling. First row illustrates the difference in relative abundance estimates between host and drag sampling at Coal Oil Point (second row = Paradise Reserve; third row = Sedgwick Reserve). Tick species: “DO” = D. occidentalis, “DV” = D. variabilis, “Hl” = H. leporispalustris, “Ib” = I. brunneus, “IP” = I. pacificus, “Ipero” = I. peromysci, and “Is” = I. spinipalpis. Some species (e.g. “IP” and “DO”) are well characterized by drag sampling, while others (e.g. “Ipero”, “Is” and “DV”) are not. (TIFF) [file pone.0201665.s012.tiff]
