# Supplementary material for: Abiotic and habitat drivers of tick vector abundance, diversity, phenology and human encounter risk in southern California
Source: PLoS One. 2018 Jul 31;13(7):e0201665. doi: 10.1371/journal.pone.0201665 (PMC6067749; doi:10.1371/journal.pone.0201665)
Supplement: S1 Table — Host sampling took place from winter of 2013 through spring of 2016 at Coal Oil Point Reserve; in winter, spring and summer of 2014 at Paradise Reserve [24]; and from winter of 2013 through winter of 2015 at Sedgwick Reserve. (DOCX) [file pone.0201665.s013.docx]

**S1 Table.**

| Reserve | # Hosts | Host Species |
| --- | --- | --- |
| Coal Oil Point | 191 | *Peromyscus maniculatus; Reithrodontomys megalotis; Microtus californicus; Mus musculus; Rattus rattus; Sceloporus occidentalis* |
| Sedgwick | 117 | *Peromyscus maniculatus; Neotoma fuscipes; Otospermophilus beecheyi; Sceloporus occidentalis; Elgaria multicarinata* |
| Paradise | 224 | *Peromyscus maniculatus; Neotoma fuscipes; Otospermophilus beecheyi; Tamias merriami; Sceloporus occidentalis; Elgaria multicarinata* |
